# Brachyury and SMAD signalling collaboratively orchestrate distinct mesoderm and endoderm gene regulatory networks in differentiating human embryonic stem cells

**DOI:** 10.1242/dev.117838

**Published:** 2015-06-15

**Authors:** Tiago Faial, Andreia S. Bernardo, Sasha Mendjan, Evangelia Diamanti, Daniel Ortmann, George E. Gentsch, Victoria L. Mascetti, Matthew W. B. Trotter, James C. Smith, Roger A. Pedersen

**Affiliations:** 1 The Anne McLaren Laboratory for Regenerative Medicine, Wellcome Trust–MRC Cambridge Stem Cell Institute, University of Cambridge, Cambridge CB2 0SZ, UK; 2 The Francis Crick Institute, Mill Hill Laboratory, The Ridgeway, London NW7 1AA, UK; 3 Department of Zoology, University of Cambridge, Cambridge CB2 3EJ, UK; 4 Department of Surgery, University of Cambridge, Cambridge CB2 0QQ, UK; 5 Cambridge Institute for Medical Research and Wellcome Trust–MRC Cambridge Stem Cell Institute, University of Cambridge, Cambridge CB2 0XY, UK

**Keywords:** SMAD, T-BOX, Embryonic stem cells, Gastrulation, Gene regulatory networks, Human

## Abstract

The transcription factor brachyury (T, BRA) is one of the first markers of gastrulation and lineage specification in vertebrates. Despite its wide use and importance in stem cell and developmental biology, its functional genomic targets in human cells are largely unknown. Here, we use differentiating human embryonic stem cells to study the role of BRA in activin A-induced endoderm and BMP4-induced mesoderm progenitors. We show that BRA has distinct genome-wide binding landscapes in these two cell populations, and that BRA interacts and collaborates with SMAD1 or SMAD2/3 signalling to regulate the expression of its target genes in a cell-specific manner. Importantly, by manipulating the levels of BRA in cells exposed to different signalling environments, we demonstrate that BRA is essential for mesoderm but not for endoderm formation. Together, our data illuminate the function of BRA in the context of human embryonic development and show that the regulatory role of BRA is context dependent. Our study reinforces the importance of analysing the functions of a transcription factor in different cellular and signalling environments.

## Abstract (precis)

**Summary:** In differentiating hESCs, BRACHYURY has cell type-specific targets and functions: it acts with SMAD1 to promote mesoderm specification, while in the endoderm it interacts with SMAD2/3.

## INTRODUCTION

The three primary germ layers (ectoderm, mesoderm and endoderm) arise from the pluripotent epiblast during gastrulation in the amniote embryo (Arnold and Robertson, 2009; Tam and Loebel, 2007); this can be modelled *in vitro* using pluripotent stem cells (Murry and Keller, 2008). The brachyury gene (*T*, *BRA*) encodes a T-box transcription factor that plays an essential role in mesoderm formation (Papaioannou, 2014; Showell et al., 2004). Brachyury is expressed first in the primitive streak during gastrulation, and later in the notochord and tailbud (Herrmann et al., 1990; Wilkinson et al., 1990). Mice lacking one copy of the gene have a short tail, while homozygous embryos die around embryonic day (E) 9-10 (Chesley, 1935). The latter develop only the first seven somites and lack a proper notochord; in addition, they display defects in left-right asymmetry and in cell migration (Showell et al., 2004). Importantly, the mutant phenotype also includes severe defects in cardiovascular and placental development (David et al., 2011; Inman and Downs, 2006; King et al., 1998). Brachyury is widely used as the earliest marker of mesodermal and also endodermal differentiation in embryonic stem cell (ESC) studies and during gastrulation, because both these cell lineages derive from the primitive streak (Murry and Keller, 2008). Indeed, definitive endoderm progenitors co-express *Bra*, *Sox17*, *Foxa2*, *Gsc* and other endoderm markers (D'Amour et al., 2005; Kubo et al., 2004; Tada et al., 2005). Based on morphological analyses, mouse *Bra* homozygotes seem to form a normal foregut but an abnormal hindgut (Chesley, 1935). However, on a molecular level, the role of BRA in endoderm formation remains poorly understood.

Genomic targets of BRA orthologues have previously been identified in zebrafish (Morley et al., 2009) and *Xenopus* embryos (Gentsch et al., 2013), and in mouse embryoid bodies (Evans et al., 2012; Lolas et al., 2014) using ChIP-chip or ChIP-seq approaches. Recently, BRA target genes were identified in differentiating hESCs (Tsankov et al., 2015), but the regulatory impact of BRA was not functionally characterized in these experiments. Thus, despite its important role in vertebrate development and its widespread use in stem cell biology (Murry and Keller, 2008; Papaioannou, 2014), the precise regulatory role of BRA and its genome-wide functional role are poorly understood in humans.

Human embryonic stem cells (hESCs) represent the best available system in which to study the molecular mechanisms underpinning human embryonic development (Murry and Keller, 2008). We have developed two *in vitro* protocols (activin or BMP4 based) (Bernardo et al., 2011) that can induce hESCs to differentiate into distinct cell populations, both expressing BRA. Strikingly, these populations have the characteristics of the anterior and posterior regions of the early primitive streak (Alev et al., 2010) from which, respectively, endoderm or mesoderm cells arise *in vivo* (Lawson et al., 1991; Parameswaran and Tam, 1995).

Here, we perform chromatin immunoprecipitation followed by high-throughput sequencing (ChIP-seq) to identify genomic targets of BRA in these two cell populations. Interestingly, these genome-wide binding events differ in activin-treated or BMP4-treated cells, suggesting that BRA interacts with the genome depending on the signalling environment and cell identity. We provide functional validation of these genomic binding events by modulating levels of BRA within different signalling environments in differentiating hESCs and by analysing the expression pattern of BRA target genes in mouse embryos that lack brachyury.

Importantly, our experiments also indicate that BRA physically interacts with downstream effectors of activin or BMP4 signalling: SMAD2/3 in endoderm progenitors and SMAD1 in mesoderm progenitors. We conclude that BMP4-SMAD1 signalling and BRA action are essential for proper mesodermal differentiation, while simultaneously repressing endodermal fates. By contrast, in partnership with eomesodermine (EOMES) and activin-SMAD2/3 signalling, BRA proves to be sufficient, but not necessary, to activate endodermal gene expression.

## RESULTS

### An *in vitro* differentiation system to study the role of BRA in human gastrulation

**Fig. 1.**
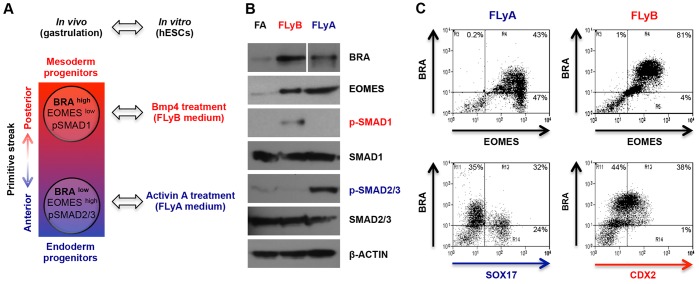
**An *in vitro* differentiation system to study the role of BRA in human gastrulation.** (A) hESCs differentiated in FLyA (blue) or FLyB (red) media for 36 h resemble the anterior (endoderm progenitors) or posterior (mesoderm progenitors) regions of the early primitive streak. (B) Western blots showing the expression of BRA, EOMES, phospho-SMAD1, total SMAD1, phospho-SMAD2/3, total SMAD2/3 and β-actin during pluripotency (FA), and in FLyA and FLyB conditions. (C) Flow cytometry analysis of hESCs differentiated in FLyA or FLyB media for 36 h. FLyA-treated cells were co-immunostained for BRA and EOMES (upper left), or for BRA and SOX17 (lower left). FLyB-treated cells were co-immunostained for BRA and EOMES (upper right), or for BRA and CDX2 (lower right).

We have previously optimised chemically defined conditions that cause hESCs to differentiate as progenitors of endoderm or of mesoderm (Bernardo et al., 2011). When hESCs are induced to differentiate (36 h of treatment with Fgf2 and the PI3 kinase inhibitor Ly294002) in an activin A-based medium (called FLyA), they upregulate endoderm markers such as *CER1*, *FOXA2*, *GSC* and *SOX17*. By contrast, hESCs similarly induced to differentiate in a BMP4-based medium (called FLyB) express mesodermal genes such as *CDX2*, *TBX6*, *FOXF1* and *BMP4* (Bernardo et al., 2011). Thus, FLyA-treated hESCs resemble the anterior region of the early primitive streak, whereas FLyB-treated hESCs resemble the posterior region (Fig. 1A) (Alev et al., 2010; Arnold and Robertson, 2009).

Significantly, these two differentiated populations express different levels of BRA and EOMES, two T-BOX transcription factors that are key regulators of gastrulation (Herrmann et al., 1990; Russ et al., 2000; Arnold et al., 2008). FLyA-treated cells are predominantly BRA^low^/EOMES^high^ and upregulate SOX17 (Fig. 1B,C; supplementary material Fig. S1A,B), whereas FLyB-treated cells are mainly BRA^high^/EOMES^low^ and upregulate CDX2 (Fig. 1B,C; supplementary material Fig. S1A,B) (Bernardo et al., 2011; Mendjan et al., 2014). As expected, activin A or BMP4 treatment triggers the phosphorylation of their downstream targets, SMAD2/3 or SMAD1, respectively (Fig. 1B). This *in vitro* differentiation system allows us to capture and analyse the transient progenitor populations that give rise to two primary embryonic tissue lineages: endoderm and mesoderm.

### Distinct genome-wide BRA-binding landscapes

**Fig. 2.**
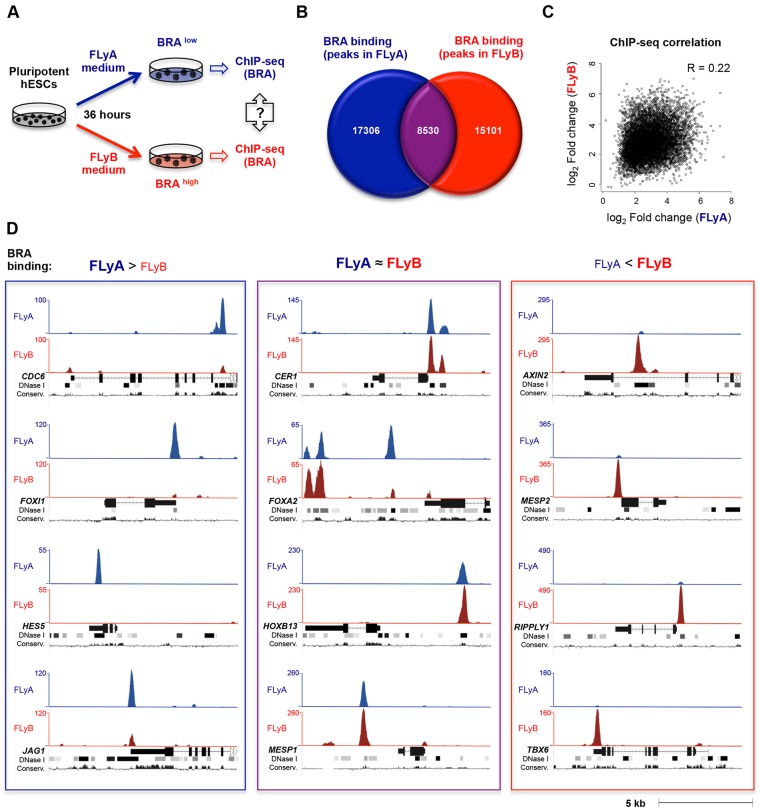
**BRA exhibits distinct genomic binding profiles in FLyA- and in FLyB-differentiated hESCs.** (A) hESCs treated with FLyA (BRA^low^) or FLyB (BRA^high^) media for 36 h were used to analyse and compare the genome-wide binding of BRA (ChIP-seq). (B) Venn diagram showing the detectable overlap between BRA binding (ChIP-seq peaks) in FLyA- and FLyB-treated hESCs. (C) Dot plot of ChIP-seq fold enrichment values (normalised to Input samples) of common BRA peaks in FLyA- and FLyB-treated hESCs. R, correlation coefficient. (D) Examples of ChIP-seq peaks depicting BRA-binding profiles in hESCs treated with FLyA (blue track) or FLyB (red track) media: stronger peaks in FLyA (left); peaks detected in both FLyA and FLyB (centre); stronger peaks in FLyB (right). Tracks under ChIP-seq peaks: gene locus (exons depicted as full rectangles, introns depicted as lines with chevrons), DNase I-hypersensitive clusters (ENCODE project) and mammalian conservation profiles (UCSC genome browser). The *y* axis shows the number of normalised unique reads.

FLyA- and FLyB-treated hESCs express, respectively, genes that are characteristic of anterior and posterior regions of the primitive streak. They also express different levels of BRA, which reaches its peak at around 36 h of differentiation (Bernardo et al., 2011). We used this differentiation system to identify genomic targets of BRA by ChIP-seq and to ask whether these differ between endoderm and mesoderm progenitors (Fig. 2A).

Two replicate ChIP-seq experiments, both with FLyA- and FLyB-treated cells, were carried out using two different anti-BRA antibodies, one from R&D Systems and the other from Santa Cruz Biotechnology. These replicates showed high correlation coefficients for BRA binding in both datasets (R=0.72 for FLyA conditions and R=0.73 for FLyB conditions) (supplementary material Fig. S2A). Overlapping ChIP-seq peaks (present using either antibody) are shown in supplementary material Table S1. However, the ChIP-seq experiments using the Santa Cruz antibody had lower signal-to-noise ratio and yielded lower ‘unique read’ counts. We therefore used the datasets generated with the R&D Systems antibody to perform all subsequent analyses.

ChIP-seq analysis indicated that there are 25,836 BRA-binding events in FLyA-treated hESCs and 23,631 in FLyB-treated hESCs (Fig. 2B). Although there is considerable overlap between these two datasets (Fig. 2B; supplementary material Fig. S2B), a large proportion of peaks are unique to each cell population: 68% in the FLyA condition and 64% in the FLyB condition (Fig. 2B). For example, BRA binds in the vicinity of genes such as: (1) *CDC6*, *FOXI1*, *HES5* and *JAG1* when cultured in FLyA but not so strongly in FLyB (Fig. 2D; left blue rectangle, FLyA>FLyB); (2) *AXIN2*, *MESP2*, *RIPPLY1* and *TBX6* when cultured in FLyB but not so strongly in FLyA (Fig. 2D; right red rectangle, FLyA<FLyB); and (3) *CER1*, *FOXA2*, *HOXB13* and *MESP1* when hESCs are cultured in FLyA or FLyB (Fig. 2D; central purple rectangle, FLyA≈FLyB). Moreover, the ‘fold enrichment’ correlation coefficient for peaks detected in both FLyA and FLyB conditions was low (R=0.22) (Fig. 2C), further underscoring the differences in BRA binding between FLyA- and FLyB-treated hESCs. Importantly, these differences in BRA binding in FLyA or FLyB conditions were confirmed by ChIP-qPCR on a set of selected targets (supplementary material Fig. S2C,D). These observations reveal that BRA has distinct genome-wide binding landscapes in hESC-derived endoderm and mesoderm progenitors.

### Developmental significance of cell type-specific BRA binding: different target genes in endoderm and mesoderm progenitors

**Fig. 3.**
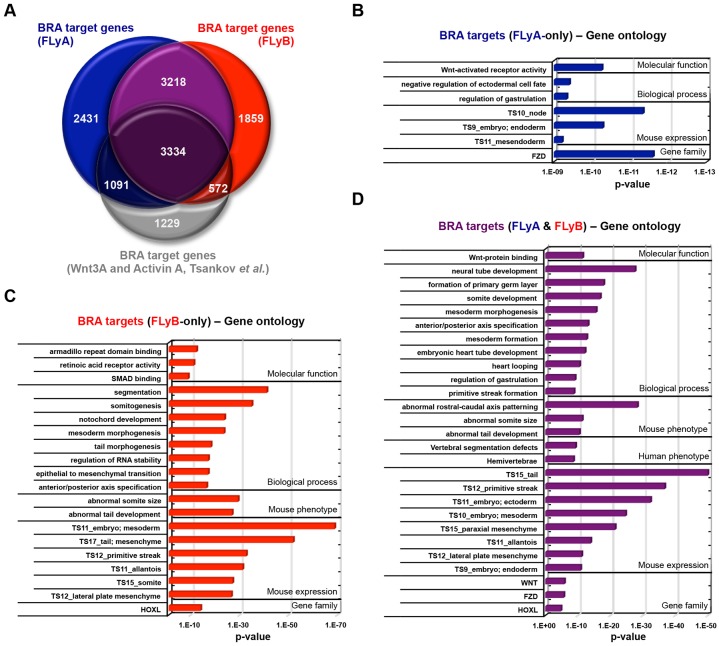
**BRA has different sets of target genes in FLyA- and FLyB-treated hESCs.** (A) Venn diagram showing the overlap of BRA putative target genes between FLyA-treated hESCs (blue), FLyB-treated hESCs (red) and WNT3A/activin- treated hESCs (grey; Tsankov et al., 2015). (B-D) Gene ontology analyses (GREAT algorithm; McLean et al., 2010) of BRA-binding regions detected only in FLyA (B), only in FLyB (C) and in both FLyA and FLyB (D). Ontology terms are ranked according to their enrichment *P*-values: ‘Gene family’ terms (*P*-value <1×10^−5^), all other terms (*P*-value <1×10^−9^). TS, Theiler stage of mouse development.

Using a ‘nearby gene’ peak annotation approach (up to 50 kb on either side of a gene), BRA binding was detected in the vicinity of 10,074 genes in FLyA-treated hESCs and 8983 genes in FLyB-treated hESCs (Fig. 3A; supplementary material Table S1). As suggested by the overlap analysis (Fig. 2A), BRA binds to many of its target genes in a cell type-specific manner. Thus, in the FLyA dataset, 35% of targets were unique to this condition and 27% were unique to the FLyB dataset (Fig. 3A). These percentages are lower than those concerning the binding overlap (Fig. 2B) because several peaks are often located around a single locus (in both promoter and enhancer regions) and because we excluded the most distal intergenic peaks, the assignment of which to the nearest gene can lead to a higher false discovery rate (data not shown).

Recently, BRA genomic binding was investigated by ChIP-seq in hESC-derived ‘mesendoderm’ progenitors (12 h treatment with WNT3A and activin A) (Tsankov et al., 2015). This dataset shows a substantial overlap with ours both in terms of binding regions (supplementary material Fig. S3A-C) and target genes (Fig. 3A). However, many unique binding events are detected in each dataset, further suggesting that the regulatory role of BRA is context dependent.

To investigate the developmental significance of our findings, we performed gene ontology analyses using GREAT (McLean et al., 2010) (supplementary material Table S2), distinguishing between FLyA-only putative targets (Fig. 3B), FLyB-only putative targets (Fig. 3C), and targets common to both FLyA and FLyB (Fig. 3D). In all subsets, there was clear enrichment for genes that are co-expressed with Bra during mouse embryogenesis and involved in all aspects of its complex mutant phenotype (Fig. 3, Table 1; supplementary material Table S2).

**Table 1.**
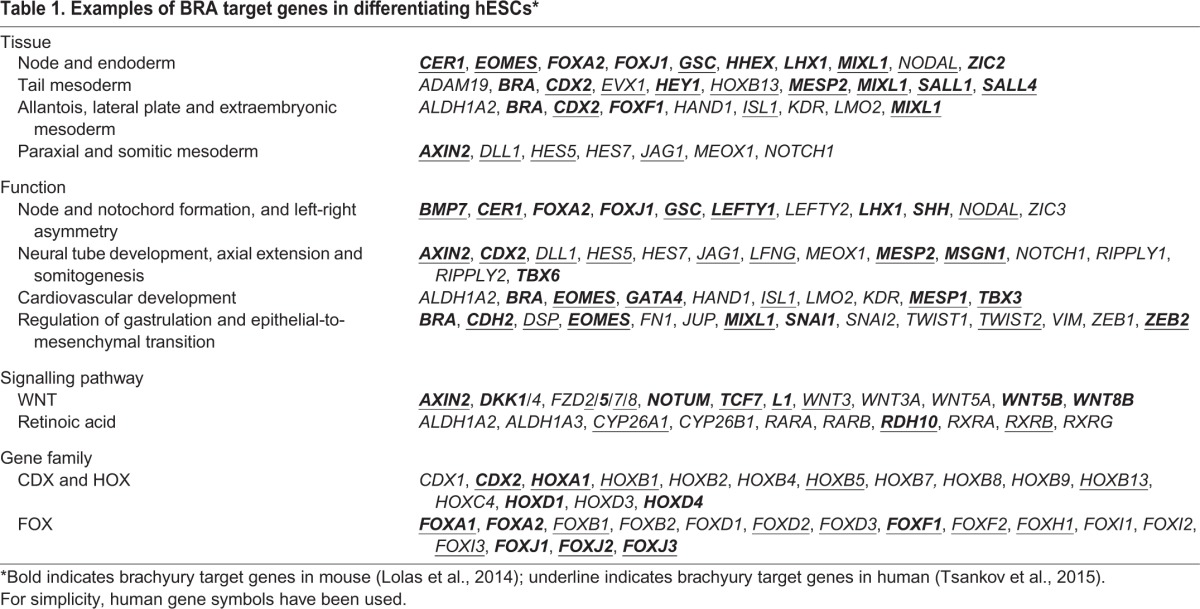
Examples of BRA target genes in differentiating hESCs*

The gene ontology analysis of the recent human dataset (Tsankov et al., 2015) included few highly significant terms (*P*-value <1×10^−9^) (supplementary material Table S2). However, the top term in the ‘mouse expression’ category was ‘TS9_primitive streak’ (*P*=4.92×10^−9^) and we note that several BRA targets that are common to the Tsankov et al. dataset and our dataset make biological sense (Table 1).

Strikingly, whereas FLyA-only BRA targets were enriched for genes expressed in anterior primitive streak derivatives, such as the node and endoderm (Fig. 3B), FLyB-only BRA targets were enriched for genes expressed in mid/posterior primitive streak derivatives, such as tail mesoderm, lateral plate mesoderm, extraembryonic mesoderm, allantois and somitic/paraxial mesoderm (Fig. 3C). BRA targets that were common to both FLyA and FLyB conditions are expressed in all germ layers (Fig. 3D).

Overall, our results show that in FLyA- and FLyB-treated cells, BRA binds to the genome in a manner that is correlated with the transcriptional and developmental identity of each cell population, endoderm and mesoderm, respectively.

### BRA genomic binding overlaps with EOMES and SMAD2/3 in endoderm progenitors

**Fig. 4.**
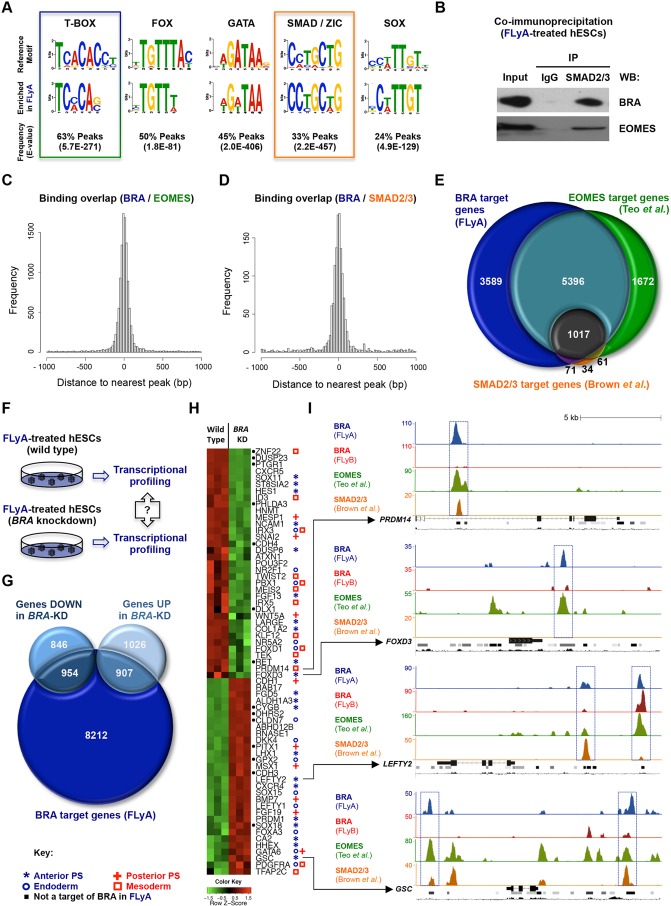
**BRA in the context of activin A signalling.** (A) Comparison of DNA recognition sites of five protein families (row above) and DNA motifs enriched at BRA FLyA ChIP-seq peaks (row below). (B) Co-immunoprecipitation of SMAD2/3 (pulldown) with BRA and EOMES (WB, western blot) in FLyA-treated hESCs; IgG (negative control immunoglobulin). (C,D) Histograms showing the distance between BRA-binding peaks in FLyA-treated hESCs and EOMES binding (Teo et al., 2011) or SMAD2/3 binding (Brown et al., 2011) in FLyAB-treated hESCs. (E) Venn diagram showing the overlap of putative target genes between BRA in FLyA-treated hESCs (FLyA, blue), EOMES (green) and SMAD2/3 (orange). (F) Wild-type (control) and *BRA* knockdown hESCs were differentiated for 36 h in FLyA and profiled for transcriptome-wide (microarray) differential expression analysis. (G) Venn diagram showing the overlap between BRA putative target genes (FLyA, dark blue) and genes that were either up- or downregulated (FDR <0.05) in *BRA* knockdown hESCs when compared with wild-type hESCs. (H) Microarray gene expression heat-map of wild-type versus *BRA* knockdown (KD) hESCs grown in FLyA for 36 h. Green indicates downregulation and red indicates upregulation. Symbols after gene names indicate expression pattern *in vivo* (Mouse Genome Informatics; Alev et al., 2010). (I) ChIP-seq peaks depicting BRA binding in hESCs treated with FLyA (blue) or FLyB (red), and EOMES binding (green) or SMAD2/3 binding (orange). Tracks under ChIP-seq peaks: gene locus (exons depicted as full rectangles, introns depicted as lines with chevrons), DNase I-hypersensitive clusters (ENCODE project) and mammalian conservation profiles (UCSC genome browser). The *y* axis shows the number of normalised unique reads. Blue boxes highlight FLyA-specific BRA binding peaks.

Having identified genomic targets of BRA in differentiating hESCs, we asked whether BRA-binding regions (200 bp sequences centred on ChIP-seq peaks) were enriched for specific DNA sequences. To this end, we performed *de novo* DNA motif analyses using the MEME suite (Bailey et al., 2009). For FLyA-treated cells, these revealed enrichment of a motif resembling the T-BOX consensus-binding sequence (Kispert and Herrmann, 1993) in 63% of peaks (Fig. 4A). Motifs for other protein families were also enriched in BRA FLyA peaks (Fig. 4A; supplementary material Fig. S4A), including FOX, GATA/GSC, SMAD/ZIC, SOX, POU and STAT, suggesting that BRA might interact with members of these families. Likely candidates, based on their expression patterns *in vivo* and in FLyA-treated cells (Alev et al., 2010; Pfister et al., 2007; Bernardo et al., 2011), include FOXA2, GATA4/6, GSC, SMAD2/3, SOX17 and POU5F1/OCT4 (supplementary material Fig. S4C,D) (Mullen et al., 2011). Significantly, we were able to show by co-immunoprecipitation that both BRA and EOMES interact with SMAD2/3 (Fig. 4B), suggesting cooperation with activin signalling. BRA-binding peaks show a remarkable overlap with those of EOMES (Teo et al., 2011) and of SMAD2/3 (Brown et al., 2011) in hESC-derived mesendodermal progenitors (Fig. 4C,D), and share with BRA many putative common target genes (Fig. 4E). Importantly, the genomic binding of EOMES and SMAD2 in FLyA-treated cells was confirmed by ChIP-qPCR on a set of selected BRA target regions (supplementary material Fig. S4B).

### BRA is largely dispensable for the expression of key endoderm markers

Both EOMES and SMAD2/3 are essential for proper expression of endoderm markers in differentiating hESCs (Brown et al., 2011; Teo et al., 2011), but little is known about the role of BRA in this context. We therefore sought to discover which putative BRA targets require normal BRA levels for their correct expression in FLyA-treated hESCs. *BRA* knockdown (shRNA KD) hESCs were compared with their wild-type counterparts by transcriptional profiling at 36 (Fig. 4F; supplementary material Table S3) and 72 h of differentiation, in more mature endoderm cells (supplementary material Fig. S4F and Table S3). At the protein level, BRA was virtually absent in *BRA* knockdown cells (supplementary material Fig. S4E). Misregulated transcripts in *BRA* knockdown cells were compared with BRA-bound genes in the FLyA condition (Fig. 4G; supplementary material Fig. S4G). Up- and downregulated genes, both at 36 and at 72 h, showed enrichment for BRA targets (*P*<0.01, Pearson's Chi-squared).

We proceeded to perform gene ontology analysis of misregulated BRA targets using GREAT (McLean et al., 2010) (supplementary material Table S4). Although BRA was necessary for the upregulation of many target genes that are developmentally important and expressed under FLyA conditions (Fig. 4H; supplementary material Fig. S4H), this subset appears to be enriched for genes expressed in neurectoderm derivatives post-gastrulation (supplementary material Table S4), which are not gene ontology categories that are developmentally relevant to FLyA-treated cells. Interestingly, however, key genes involved in epithelial-to-mesenchymal transition (EMT) were downregulated at 36 h (*SNAI2*, *TWIST2* and *FOXD3*), together with the upregulation of *CDH1*/E-cadherin, a classical hallmark of impaired EMT (Lamouille et al., 2014).

Strikingly, the expression of many genes expressed in the anterior primitive streak and involved in endoderm formation was either unaltered or even upregulated in *BRA* knockdown cells (*P*=3.49×10^−11^ at 36 h, *P*=1.48×10^−11^ at 72 h; supplementary material Table S4). The latter included genes such as *GSC*, *GATA6*, *HHEX*,* LEFTY1/2*, *CXCR4*, *OTX2* and *LHX1* (Fig. 4H; supplementary material Fig. S4H). These observations were confirmed by qRT-PCR in another *BRA* knockdown clone (81% *BRA* knockdown efficiency in FLyA treatment at 36 h) when compared with a scrambled (mock shRNA) control line (supplementary material Fig. S4I). Under FLyA conditions, many of these genes were not only bound by BRA but also by EOMES and SMAD2/3 (Fig. 4I).

Together, these data show that the genome-wide binding of BRA in endoderm progenitors broadly overlaps with that of EOMES and SMAD2/3, both of which are essential regulators of endoderm formation. However, BRA is not necessary for the expression of most key endoderm markers.

### BRA genomic binding overlaps with EOMES and SMAD1 in mesoderm progenitors

**Fig. 5.**
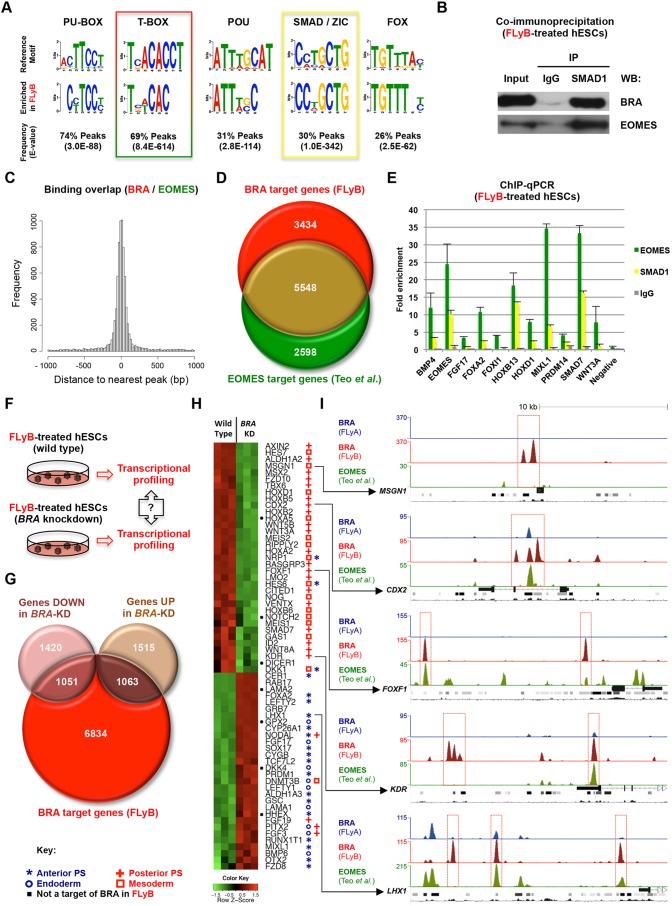
**BRA in the context of BMP4 signalling.** (A) Comparison of DNA recognition sites of five protein families (row above) and DNA motifs enriched in BRA FLyB ChIP-seq peaks (below). (B) Co-immunoprecipitation of SMAD1 (pulldown) with BRA and EOMES (western blot) in FLyB-treated hESCs; IgG (negative control immunoglobulin). (C) Histogram showing the distance between BRA-binding peaks in FLyB-treated hESCs and EOMES binding (Teo et al., 2011) in FLyAB-treated hESCs. (D) Venn diagram showing the overlap of putative target genes between BRA in FLyB-treated hESCs (FLyB, red) and EOMES (green). (E) Graph with fold enrichment values (ChIP over input) for EOMES binding (green), SMAD1 binding (yellow) and control IgG binding (grey) to BRA target regions in FLyB-treated hESCs (36 h). Error bars correspond to s.d. (*n*=3). ChIP-qPCR values were normalised to the highest control IgG value (*PRDM14*). (F) Wild-type (control) and *BRA* knockdown hESCs were differentiated for 36 h in FLyB and profiled for transcriptome-wide (microarray) differential expression analysis. (G) Venn diagram showing the overlap between BRA putative target genes (FLyB, red) and genes that were either up- or downregulated (FDR <0.05) in *BRA* knockdown hESCs when compared with wild-type hESCs. (H) Microarray gene expression heat-map of wild-type versus *BRA* knockdown (KD) hESCs grown in FLyB for 36 h. Green indicates downregulation and red indicates upregulation. Symbols after gene names indicate expression pattern *in vivo* (Mouse Genome Informatics; Alev et al., 2010). (I) ChIP-seq peaks depicting BRA binding in hESCs treated with FLyA (blue) or FLyB (red), and EOMES binding (green). Tracks under ChIP-seq peaks: gene locus (exons depicted as full rectangles, introns depicted as lines with chevrons), DNase I-hypersensitive clusters (ENCODE project), mammalian conservation profiles (UCSC genome browser). The *y* axis shows the number of normalised unique reads. Red boxes highlight FLyB-specific BRA binding peaks.

We also performed *de novo* DNA motif analysis (Bailey et al., 2009) using the FLyB BRA peak dataset. As observed with the FLyA dataset, this analysis revealed enrichment (69% of peaks) of the T-BOX consensus binding sequence (Kispert and Herrmann, 1993) (Fig. 5A). Motifs characteristic of other protein families were also found in BRA FLyB peaks (Fig. 5A; supplementary material Fig. S5A), including PU-BOX, POU, SMAD/ZIC, FOX, KLF and GATA, again suggesting that BRA might interact with members of these families. Likely candidates, based on their expression patterns *in vivo* and in FLyB-treated cells (Alev et al., 2010; Pfister et al., 2007; Bernardo et al., 2011), include SMAD1, FOXF1, GATA2/3 and POU5F1/OCT4 (supplementary material Fig. S5B,C) (Mullen et al., 2011). Previous work has shown that a BRA orthologue, Xbra, directly interacts with Smad1 in *Xenopus* embryos (Messenger et al., 2005). Significantly, we were able to show by co-immunoprecipitation that both BRA and EOMES interact with SMAD1 (Fig. 5B), suggesting cooperation with BMP4 signalling. BRA binding peaks also showed close proximity to those of EOMES in hESC-derived mesendodermal progenitors (Teo et al., 2011) (Fig. 5C), with a substantial number of common putative target genes (Fig. 5D). Importantly, the genomic binding of EOMES and SMAD1 in FLyB-treated cells was confirmed by ChIP-qPCR on a set of selected BRA target regions (Fig. 5E).

### BRA is necessary for mesoderm gene expression

We next asked which putative BRA targets require normal BRA levels for their correct expression in BMP4-treated cells by comparing the transcriptomes of *BRA* knockdown (shRNA KD) hESCs with their wild-type counterparts at 36 h of FLyB differentiation (Fig. 5F; supplementary material Table S3) and at 72 h of FLyB/FB differentiation (supplementary material Fig. S5D and Table S3), when cells resemble extraembryonic and lateral plate mesoderm (Bernardo et al., 2011; Cheung et al., 2012; Mendjan et al., 2014). Misregulated transcripts in *BRA* knockdown cells were compared with BRA-bound genes in the FLyB condition (Fig. 5G; supplementary material Fig. S5E). Up- and downregulated genes, both at 36 and 72 h, showed enrichment for BRA targets (*P*<0.01, Pearson's Chi-squared).

We then proceeded to perform gene ontology analysis of misregulated BRA targets using GREAT (McLean et al., 2010) (supplementary material Table S4). Significantly, BRA was necessary for the normal upregulation of many genes expressed in BMP4-treated cells (at 36 and 72 h) that are essential for mesoderm development. These include *ALDH1A2* (*RALDH2*), *AXIN2*, *CDX2*, *FOXF1*, *KDR*,* LMO2*, *MSGN1*, *MEIS1*, *TBX6* and *WNT3A* (Fig. 5H; supplementary material Fig. S5F). Indeed, gene ontology analysis shows enrichment for phenotypes that are reminiscent of *BRA* mutant embryos, such as ‘abnormal somite size’ (*P*=9.26×10^−24^), ‘abnormal tail development’ (*P*=1.90×10^−22^), ‘abnormal gastrulation’ (*P*=1.56×10^−17^) and ‘abnormal vascular development’ (*P*=3.29×10^−16^) (supplementary material Table S4). Interestingly, several HOX genes were also downregulated upon *BRA* knockdown at 36 h (Fig. 5H) (*P*=1.30×10^−12^; supplementary material Table S4), an effect that was even more marked at 72 h of differentiation (supplementary material Fig. S5F) (*P*=3.26×10^−6^; supplementary material Table S4).

Noticeably, the expression of many endoderm regulators was upregulated in *BRA* knockdown cells grown in FLyB conditions (*P*=3.87×10^−19^ at 36 h, *P*=8.36×10^−16^ at 72 h; supplementary material Table S4). These include *CER1*, *CYP26A1*, *EOMES*, *FOXA2*, *GSC*, *GATA6*, *HHEX*, *LEFTY1/2*, *LHX1*, *MIXL1*, *OTX2* and *SOX17* (Fig. 5H; supplementary material Fig. S5F). These observations were confirmed by qRT-PCR in another *BRA* knockdown clone (89% *BRA* knockdown efficiency in FLyB treatment at 36 h) when compared with a scrambled (mock shRNA) control line (supplementary material Fig. S5G). Strikingly, BRA was bound in the vicinity of some of these genes in regions only detected in the FLyB condition (e.g. *LHX1*, Fig. 5I).

Together, these data show that the genome-wide binding of BRA in mesoderm progenitors overlaps with that of EOMES. However, unlike EOMES (Teo et al., 2011), BRA was necessary for the expression of many genes involved in mesoderm formation, while simultaneously repressing the expression of endoderm markers.

### BRA cooperates with activin or BMP4 signalling to upregulate endoderm or mesoderm markers

**Fig. 6.**
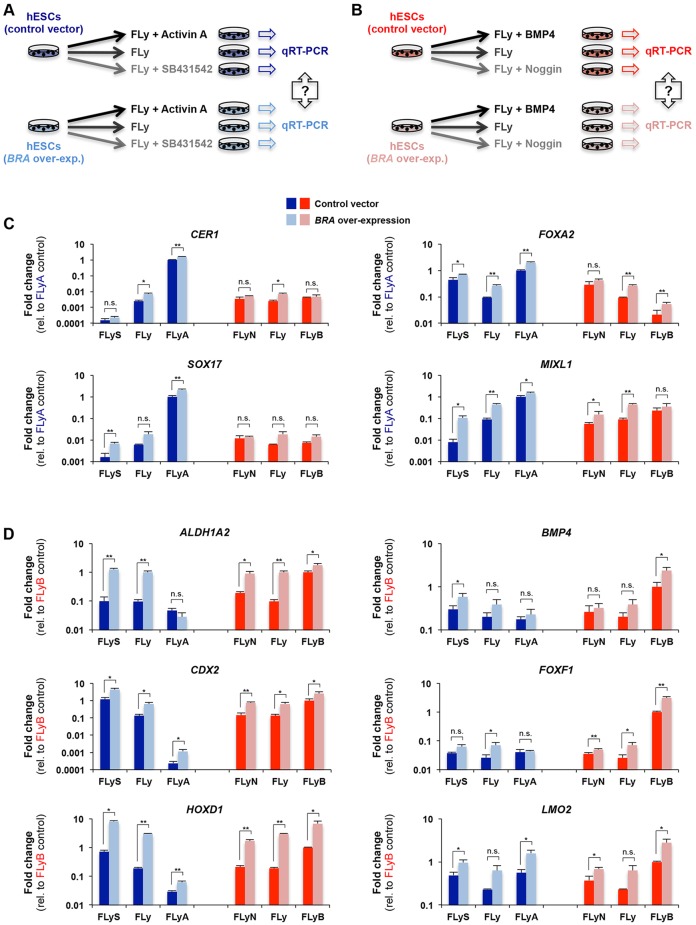
**BRA depends on the signalling environment to regulate key developmental genes.** (A,B) hESCs transfected with either an empty/control vector or a *BRA* over-expression vector were differentiated for 36 h, as indicated and samples were collected to perform comparative gene expression analysis by qRT- PCR. (C,D) qRT-PCR of BRA target genes expressed in endoderm/anterior primitive streak (blue) or in mesoderm/posterior primitive streak (red), respectively. hESCs transfected with either an empty vector (dark colours) or a *BRA* overexpression vector (light colours) were differentiated as indicated in A,B. For each group of germ layer markers, gene expression is presented as fold change over the wild type reference sample (control vector): FLyA for endoderm genes (C) and FLyB for mesoderm genes (D), as indicated on the *y* axis. Bars indicate s.d. (*n*=3) (Student's two-tailed *t*-test: *P<0.05; **P<0.01; n.s., not significant). F, FGF2; Ly, Ly294002; B, BMP4; A, activin A; N, noggin (N); S, SB431542.

Having established that *BRA* is required for the expression of many mesodermal but not endodermal genes, we asked whether BRA overexpression (*BRA* OE) (supplementary material Fig. S6A,B) in hESCs was sufficient to up- or downregulate its genomic targets. Bearing in mind that BRA has different targets in activin- or BMP4-treated cells, we analysed the phenotype of control and *BRA* OE hESC subclones in different signalling environments. All cells were grown in the presence of Fgf2 and Ly294002 as a basal differentiation medium (FLy) with the addition of activin (FLyA), BMP4 (FLyB), SB431542 (FLyS) to block activin-SMAD2/3 signalling or noggin (NOG) (FLyN) to block BMP4-SMAD1 signalling (Fig. 6A,B).

Overexpression of BRA caused the upregulation of both endodermal (*CER1*, *FOXA2*, *SOX17*, *MIXL1*) and mesodermal (*ALDH1A2*, *BMP4*, *CDX2*, *FOXF1*, *HOXD1*, *LMO2*) target genes. Strikingly, however, strong upregulation of genes characteristic of endoderm/anterior primitive streak required activin signalling (Fig. 6C). Genes characteristic of mesoderm/posterior primitive streak either required or benefitted from BMP4 signalling and, interestingly, in some cases, activin antagonism (Fig. 6D). These experiments reveal that BRA expression alone is not sufficient to fully upregulate some of its target genes to wild-type levels and that it requires appropriate signalling cues to activate the expression of its genomic targets.

### BRA target genes in the mouse

**Fig. 7.**
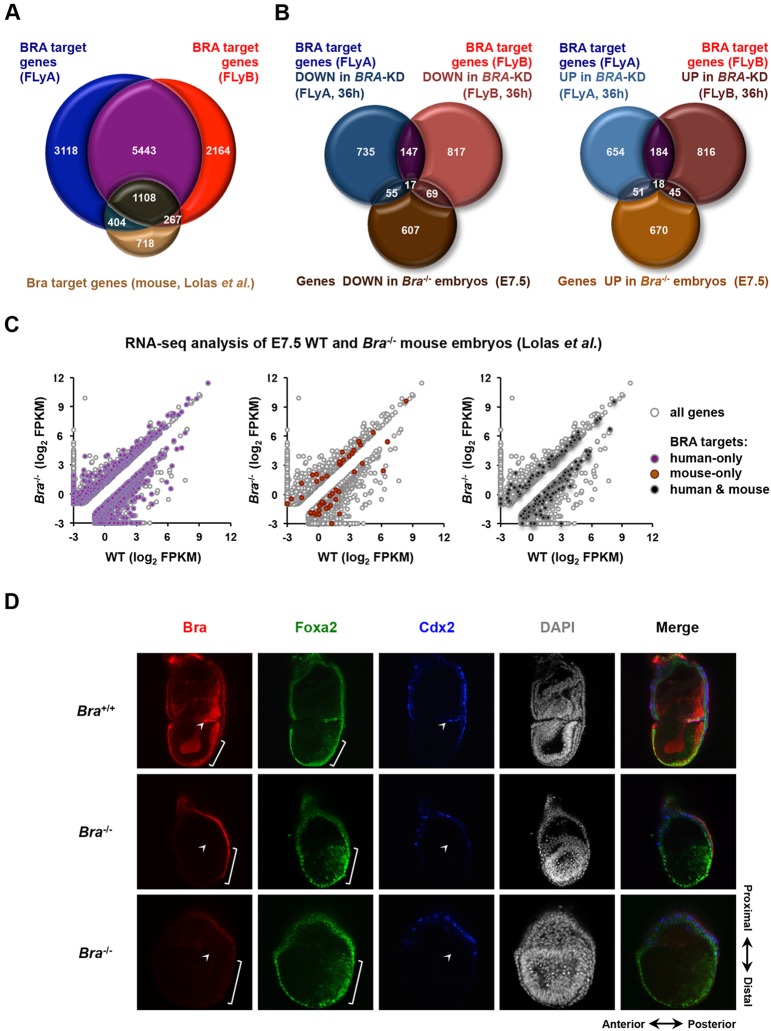
**BRA target gene expression in mouse embryos.** (A) Venn diagram showing the overlap between BRA putative target genes identified in FLyA-treated hESCs (blue), FLyB-treated hESCs (red) and activin-treated mouse embryoid bodies (light brown, Lolas et al., 2014). (B) Venn diagrams showing the overlap between BRA putative target genes that are misregulated in FLyA- or FLyB-treated *BRA* knockdown hESCs at 36 h and genes misregulated in E7.5 *Bra*^−/−^ mouse embryos (Lolas et al., 2014). Left diagram, downregulated genes; right diagram, upregulated genes. (C) RNA-seq analysis of E7.5 wild-type and *Bra*^−/−^ mouse embryos (Lolas et al., 2014); coloured dots indicate BRA target genes identified only in human (FLyA and FLyB datasets, purple), only in mouse (Lolas et al., brown) or in both human and mouse (black). Scale represents log_2_ FPKM (fragments per kilobase of exon per million fragments mapped). Only differentially expressed (fold change >2) genes are shown. (D) Confocal microscopy analysis (middle embryo stack) of mouse gastrulae (E7.0) immunostained for Bra (red), Foxa2 (green) and Cdx2 (blue). Nuclei were stained with DAPI. Upper row shows a wild-type embryo (*Bra*^+/+^). Bottom rows show *Bra* mutant embryos (*Bra*^−/−^). Spatial orientation of the embryos is shown in the lower right corner.

BRA putative target genes have been identified by ChIP-seq in activin-treated mouse embryoid bodies (Lolas et al., 2014). Our analysis reveals a considerable overlap between our datasets and that of Lolas et al., as 71% of the mouse targets are included in the human datasets (Fig. 7A). Indeed, many of these conserved targets make biological sense (Table 1). Importantly, our datasets also reveal the identity of BRA targets not identified in mouse (Table 1), including *ALDH1A2*, *HES7*, *KDR*, *LMO2* and *MEOX1*.

In order to investigate whether our observations made with *BRA* knockdown hESCs *in vitro* are also relevant *in vivo*, we analysed the expression of BRA targets in mouse *Bra* mutants compared with wild-type embryos at E7.5-8.0 (Fig. 7B; supplementary material Table S5) (fold change>2; RNA-seq data from Lolas et al., 2014). E7.5 is a comparable developmental stage to our 36 h hESC differentiation protocol. These analyses show that orthologues of key BRA mesodermal targets such as *Aldh1a2*, *Cdh2* (N-cadherin), *Foxd3*, *Hes7*, several Hox genes, *Msgn1*, *Tbx4*, *Tbx6* and *Wnt5b* are downregulated in *Bra*^−/−^ mouse embryos (supplementary material Table S5). In parallel, targets such as *Cdh1*, *Cer1* and *Eomes* are upregulated in *Bra*^−/−^ mouse embryos (supplementary material Table S5). Other anterior/endoderm markers, including *Cyp26a1*, *Foxa2*, *Gata6*, *Otx2* and *Sox17*, are also upregulated in *Bra*^−/−^ mouse embryos but to a lesser extent (fold change <2). More broadly, 823 putative BRA targets exclusively found in the human ChIP-seq datasets show misregulation in mouse mutant embryos (Fig. 7C), underscoring the value of our new data.

However, RNA-seq data analysis does not provide information on spatial expression patterns *in vivo*. Thus, we analysed the expression pattern of two key BRA target genes at the protein level in both wild-type and *Bra* mutant mouse embryos: the mesoderm regulator *Cdx2* (Chawengsaksophak et al., 2004) and the endoderm regulator *Foxa2* (Ang and Rossant, 1994; Weinstein et al., 1994; Burtscher and Lickert, 2009). In *Bra*-null embryos, *Cdx2* mRNA is downregulated 1.22 fold, while *Foxa2* mRNA is upregulated 1.30 fold when compared with wild type (Lolas et al., 2014).

In agreement with our prediction, Cdx2 nuclear expression, which is visible in a small group of cells in the proximal primitive streak where extraembryonic mesoderm is being formed (*Bra*^+/+^, white arrowhead, Fig. 7D) (Beck et al., 1995) is completely lacking in *Bra* mouse mutants (*Bra*^−/−^, white arrowheads, Fig. 7D). Foxa2 expression, which marks the distal (future anterior) region of the primitive streak (*Bra*^+/+^, white bracket, Fig. 7D) and is also expressed in extraembryonic endoderm (Burtscher and Lickert, 2009) is clearly present in *Bra*-null embryos (*Bra*^−/−^, white brackets, Fig. 7D; supplementary material Fig. S7A), and is seemingly upregulated in the mid/proximal primitive streak. Together, the results obtained with mouse embryos that lack Bra are consistent with our findings in differentiating hESCs: BRA upregulates key mesoderm genes and is dispensable for the expression of several endoderm markers.

## DISCUSSION

### BRA participates in distinct gene regulatory networks in different cell lineages and species

BRA ChIP-seq analysis of hESCs differentiating in FLyA-, FLyB- or WNT3A- and activin-containing media (Tsankov et al., 2015) showed similar binding sites but also significantly different DNA occupancy levels of BRA. Chromatin accessibility is suggested to be a prerequisite for many sequence-specific transcription factors such as BRA to directly bind DNA (Biggin, 2011; Voss and Hager, 2014). Thus, differences in BRA binding may arise from differential nucleosome occupancy, distinct histone modifications, or the presence or absence of specific protein partners (Biggin, 2011; Spitz and Furlong, 2012; Voss and Hager, 2014). It may be that activin, BMP4 and WNT3A signalling cascades interact with chromatin remodelling complexes (van Grunsven et al., 2005) so as to affect DNA accessibility in a cell type-specific manner.

It is unlikely that the distinct BRA binding in FLyA or FLyB occurs solely because BRA protein levels are lower in hESCs cultured in FLyA rather than FLyB, as there are many binding sites in FLyA-cultured cells with equal or even higher occupancy levels than in FLyB-culture cells. Furthermore, the strong correlation of BRA differential binding with the distinct transcriptional identities of FLyA- or FLyB-treated cells suggests that these events are biologically meaningful. For example, in the FLyB condition (BRA^high^), BRA binds close to many genes involved in posterior mesoderm development, consistent with fate-mapping studies showing that this tissue emerges from the posterior primitive streak, where Bra levels are higher (Lawson et al., 1991; Wilson and Beddington, 1997). Gene ontology analysis reveals that BRA binds in the vicinity of genes involved in specific developmental contexts and that are expressed in tissues where BRA function is essential. These contexts include: epithelial-to-mesenchymal transition; node and notochord formation; the establishment of left-right asymmetry (including heart looping), axial extension and somitogenesis; and cardiovascular development (Fig. 3).

Interestingly, the most highly enriched category in FLyA and FLyB common peaks was ‘neural tube development’. Indeed, BRA seems to repress neurectoderm genes in the tail bud ‘stem cell’ pool that gives rise to posterior structures such as somites, the notochord and the neural tube (Martin and Kimelman, 2010; Gentsch et al., 2013; Tzouanacou et al., 2009; Wilson et al., 2009).

We observed a substantial overlap between BRA targets identified in FLyA- or FLyB-treated hESCs and those identified in WNT3A/activin-treated hESCs (Tsankov et al., 2015) or identified in activin-treated mouse embryoid bodies (Lolas et al., 2014) – the latter suggesting a considerable degree of evolutionary conservation. The fact that the BRA targets we uncovered differ from those of Tsankov et al. could be due to distinct signalling environments, developmental stages, ChIP protocols, antibodies used or other technical issues. We note that both in Tsankov et al. and in Lolas et al. the authors used activin treatment to induce ESC differentiation, thus probably enriching for endoderm progenitors. Indeed, their datasets show a greater overlap with FLyA treatment, rather than with FLyB. Possibly due to this, Tsankov et al. and Lolas et al. did not detect BRA binding in the vicinity of many important mesodermal loci (Table 1). These include key genes in posterior mesoderm and cardiovascular development such as *ALDH1A2*, *HAND1*, *HES7*, *KDR*, *LMO2* and *MEOX1*, which thus seem to be human specific and unique to our datasets. In summary, our ChIP-seq datasets, together with those recently generated (Lolas et al., 2014; Tsankov et al., 2015), have greatly expanded our knowledge of BRA targets in mammals and now provide a rich resource for future studies to discover new regulators of human gastrulation.

### BRA and its potential protein interactors

In addition to the T-BOX consensus binding motif (T-site), DNA motif analyses revealed enrichment for binding sites for protein families with important roles during embryonic development such as FOX, POU, SOX and GATA. Of the many motifs enriched in BRA-binding sites, we focused on the SMAD/ZIC motif (Yoon et al., 2011). SMAD proteins are frequently recruited by master transcription factors to regulate cell type-specific gene regulatory programs (Mullen et al., 2011). Indeed, we found that BRA interacts with SMAD2/3 in FLyA-treated cells and with SMAD1 in FLyB-treated cells. Overexpression of BRA in hESCs upregulates several mesodermal and endodermal target genes, but this effect is particularly potent in the presence of either BMP4 or activin in the culture medium, thus reinforcing the idea that BRA collaborates with these SMAD signalling cascades to regulate its targets in a cell type-specific manner.

BRA binding in FLyA-treated hESCs overlaps very significantly with EOMES binding (Teo et al., 2011; Tsankov et al., 2015). Like other T-BOX proteins in zebrafish (Bra, Ntl, Spt and Tbx6) (Wardle and Papaioannou, 2008), in *Xenopus* (Xbra, Xbra3, VegT and Eomes) (Gentsch et al., 2013) or in the mouse (Bra and Eomes) (Costello et al., 2011, David et al., 2011), human EOMES and BRA might be functionally interconnected in distinct developmental contexts.

Interestingly, the POU core motif was also enriched in BRA peaks. Although OCT4 is traditionally regarded as a pluripotency regulator, it also acts as lineage specifier (Loh and Lim, 2011; Thomson et al., 2011; Wang et al., 2012). Indeed, OCT4 is expressed throughout the primitive streak during gastrulation (Downs, 2008), together with Bra. Although we (data not shown) and others (Pereira et al., 2011) have been unable to detect a direct physical interaction between BRA and OCT4, it is tempting to speculate that BRA might cooperate with OCT4 and/or other pluripotency factors during the early stages of gastrulation by co-regulating some common target genes, possibly through dynamic sequential binding to these loci (Spitz and Furlong, 2012; Voss and Hager, 2014). Recent ChIP-seq data analysis seems to corroborate this hypothesis (Tsankov et al., 2015).

### Searching for functional targets of BRA

Comparison of our ChIP-seq data with results obtained from transcriptional profiling of *BRA* knockdown cells showed that, although significantly enriched for BRA targets, around half of up- and downregulated transcripts do not appear to be direct targets of BRA and, conversely, that the expression of most putative direct targets is not affected by loss of BRA function. This outcome may derive in part from the inefficiency of our shRNA approach to completely eliminate BRA (supplementary material Fig. S4E), but it is also likely to reflect the functional complexity of transcriptional networks (Biggin, 2011; Spitz and Furlong, 2012). For example, BRA shares a vast number of targets with EOMES. This complicates the analysis of BRA-driven gene regulatory networks because a redundancy with EOMES might mask the number of functional target genes of BRA, as seen in *Xenopus* embryos (Gentsch et al., 2013).

The number of functional targets of BRA might well be extended if *BRA* knockdown hESCs were tested in other differentiation protocols where BRA has proposed functions, including primordial germ cells (Aramaki et al., 2013), axial mesoderm (Winzi et al., 2011), and paraxial and cardiac mesoderm (Mendjan et al., 2014). Indeed, our BRA ChIP-seq data revealed that BRA binds in the vicinity of genes involved in the formation of these lineages (Fig. 3).

Importantly, the comparison of our human datasets with mouse Bra ChIP-seq data and RNA-seq from *Bra*-null gastrulae (Lolas et al., 2014) provides *in vivo* validation for key BRA targets and underscores the novelty of our work, because around 800 BRA putative targets exclusively identified in our ChIP-seq datasets are either down- or upregulated in *Bra* mutant embryos (Fig. 7C).

### BRA as an essential mesoderm inducer and as an apparent endoderm repressor

Gene ontology analysis of transcriptional changes for BRA target genes observed in BMP4-treated *BRA* knockdown cells revealed a clear phenotypic scenario: while several BRA target genes involved in mesoderm formation were markedly downregulated, many genes important for endoderm development were upregulated. In agreement with our results, it has been shown that Bra, in collaboration with Mixl1, is able to repress endoderm or anterior primitive streak markers such as *Gsc* and *Pdgfra* (Pereira et al., 2011). However, the mechanism by which BRA regulates endoderm differentiation is likely to be complex, because elevated levels of BRA in FLyA-treated cells cause an increase in expression of endodermal marker genes (Fig. 6). Similar results have been obtained by Kalisz et al. (2012), who showed that *BRA* knockdown and *BRA* overexpression both cause upregulation of the endodermal markers GSC and SOX17 in hESCs.

Interestingly, the forced expression of several *BRA* orthologues in *Xenopus* animal caps leads to mesoderm but not to endoderm formation, except when using the *Drosophila* and *Ciona* orthologues (Marcellini et al., 2003). In these two organisms, the N-terminal domain of *Bra*, which is essential for the interaction with SMAD1 (Messenger et al., 2005), is not conserved. Indeed, a truncated form of Xbra, which can bind DNA but is unable to interact with SMAD1, can induce the expression of Gsc, an anterior/endoderm marker (Messenger et al., 2005). These observations suggest that *Bra* induces mesoderm formation when cooperating with SMAD1, and endoderm formation when the interaction with SMAD1 is absent (Marcellini, 2006). This may account for the aforementioned paradox that BRA can both repress and activate endoderm gene expression.

By contrast, gene ontology analysis of downregulated transcripts in activin-treated *BRA* knockdown cells at either 36 or 72 h of differentiation did not reveal any significant biological insights into the role of BRA as an activator in FLyA-treated cells. However, one important aspect that emerged from this profiling was the observation that genes classically involved in EMT were misregulated in FLyA-treated *BRA* knockdown cells and also *in vivo* (Lolas et al., 2014). This provides a new molecular insight into the known migratory defects of *Bra* knockout cells (Yanagisawa et al., 1981) and agrees with the EMT-promoting role of BRA in human cancer samples (Fernando et al., 2010). Indeed, the focus on BRA as an important player in cancer biology, particularly in chordoma, has been growing over the past decade (Papaioannou, 2014; Nelson et al., 2012).

### A model for BRA-mediated cell fate decisions in the primitive streak

**Fig. 8.**
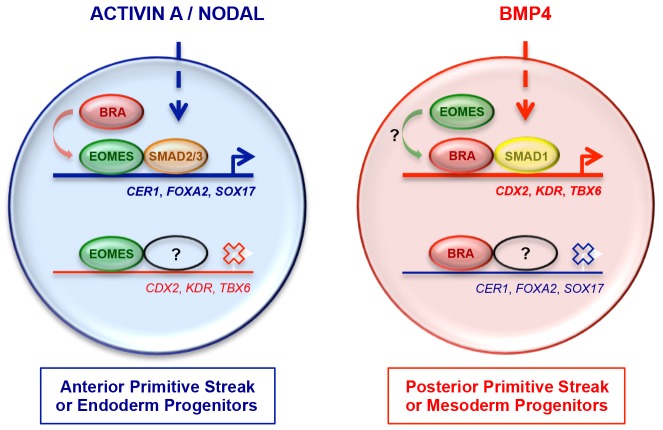
**BRA, EOMES and SMAD signalling mediate mesoderm or endoderm cell fate choice during gastrulation.** Simplified model of gene regulatory mechanisms operating in cells of the anterior (blue) or posterior (red) early primitive streak. Dashed arrows indicate activin A or NODAL as upstream activators of SMAD2/3, and BMP4 as the upstream activator of SMAD1. Crosses over white arrows indicate transcriptional silencing, whereas coloured arrows indicate transcriptional activation.

Our results provide the basis for the following model explaining the establishment of the gene expression patterns in the primitive streak that result in cells acquiring different fates (Fig. 8). Anterior early primitive streak cells, which give rise to definitive endoderm and axial mesoderm (Lawson et al., 1991), experience high levels of NODAL signalling (Tam and Loebel, 2007; Arnold and Robertson, 2009). This induces the expression of high levels of EOMES (Arnold et al., 2008; Teo et al., 2011) and low levels of BRA (Bernardo et al., 2011; Huber et al., 2004) (Fig. 1). EOMES then cooperates with NODAL-SMAD2/3 signalling to induce the expression of anterior markers such as *CER1*, *FOXA2* and *SOX17*, while repressing posteriorly expressed genes (Brown et al., 2011; Teo et al., 2011). Interestingly, by targeting the same genomic locations as EOMES, BRA is also able to activate the expression of endodermal genes, by cooperating with SMAD2/3 (Fig. 4). However, BRA is not required for anterior primitive streak gene expression (Fig. 4) where EOMES is the main player (Arnold et al., 2008; Teo et al., 2011).

At the opposite end of the early primitive streak, which gives rise to extraembryonic and posterior mesoderm (Lawson et al., 1991; Parameswaran and Tam, 1995), cells experience high levels of BMP4 signalling (Tam and Loebel, 2007; Arnold and Robertson, 2009). This induces high levels of BRA and low levels of EOMES (Bernardo et al., 2011; Huber et al., 2004) (Fig. 1). BRA, in cooperation with BMP4-SMAD1 signalling (Marcellini, 2006; Messenger et al., 2005), then induces the characteristic expression of posterior markers such as HOX genes (Wacker et al., 2004), *CDX2*, *TBX6* and *KDR* (Huber et al., 2004), while repressing the expression of anterior genes (Fig. 5). Whether EOMES can activate mesodermal gene expression in collaboration with BMP4-SMAD1 signalling remains an unresolved issue.

Together, our findings illuminate the function of BRA in the human species. We have shown that BRA is indeed both necessary and sufficient to regulate the transcription of many of its putative targets, which are key players during mesoderm or endoderm development. Moreover, our study shows that the regulatory role of BRA is context dependent, thus establishing an intimate collaboration of BRA with SMAD2/3 in an activin/NODAL-dominated context and with SMAD1 in a BMP4-dominated context.

Our study thus reinforces the value of hESCs as tools to model human embryonic development (Murry and Keller, 2008) and emphasizes the importance of analysing the functions of individual members of complex transcription factor networks in distinct cellular and signalling contexts (Spitz and Furlong, 2012).

## MATERIALS AND METHODS

### Human ESC culture in chemically defined conditions

Human ESCs (H9 line, WiCell) were grown in a chemically defined medium (CDM) as previously described (Bernardo et al., 2011; Vallier and Pedersen, 2008). For differentiation, cells were grown in CDM containing PVA instead of BSA and supplemented as described in the text and in the methods in the supplementary material. Transfection and selection of stable knockdown or overexpression clones were carried out as described in the methods in the supplementary material.

### RNA extraction, cDNA synthesis and quantitative PCR

Total RNA was extracted using the RNeasy Mini kit (Qiagen). Half a microgram of RNA was reverse transcribed using the Maxima First Strand cDNA Synthesis Kit for RT-qPCR (Thermo Scientific). Quantitative reverse transcription polymerase chain reaction (qRT-PCR) mixtures were prepared using Fast SYBR Green Master Mix (Applied Biosystems). PCR reactions were performed in a 7500 Fast Real-Time PCR System (Applied Biosystems). All procedures followed manufacturer's instructions. See also methods and Table S6 in the supplementary material.

### Co-immunoprecipitation and western blotting

Nuclear extract preparation, co-immunoprecipitation and western blotting are described in the methods in the supplementary material.

### Immunofluorescence

Cells were fixed for 10 min at room temperature in 4% paraformaldehyde and immunostained as described in the methods in the supplementary material. Fluorescent images were taken using an Olympus IX71 microscope.

### Flow cytometry of intracellular proteins

Cells were fixed and immunostained using the Cytofix-Cytoperm kit (BD Biosciences) as described in the methods in the supplementary material. Cells were analysed using a Beckman Coulter CyAn_ADP_ flow cytometer and FlowJo software (Becton Dickinson).

### ChIP-seq analysis

Chromatin immunoprecipitation was performed as previously described (Brown et al., 2011), with some modifications. Sequencing libraries were prepared using the ChIP-seq DNA sample kit (Illumina, IP-102-1001) with some modifications and sequenced with a Genome Analyzer II (Illumina). Data were further processed using the MACS peak finder (Zhang et al., 2008). DNA motifs were analysed using the MEME suite (Bailey et al., 2009). ChIP-seq data were visualised using the UCSC Genome Browser (Kent et al., 2002). Gene ontology analysis was performed using GREAT (McLean et al., 2010) with default parameters. Data are available in the GEO database (www.ncbi.nlm.nih.gov/geo) under accession number GSE60606. See methods in the supplementary material for further details.

### Microarray analysis

Sample preparation was performed according to manufacturer's instructions (Illumina). Labelled extracts were hybridised to whole-genome bead array (HumanWG-6 v3.0 Expression BeadChip) on an Illumina BeadArray reader. Gene expression heat-maps were generated by importing subsets of processed microarray data as described in the methods in the supplementary material. Data are available in the ArrayExpress database (www.ebi.ac.uk/arrayexpress) under accession numbers E-MTAB-2912 and E-MTAB-464.

### Mouse embryo work

T/+×T/+ mice (King et al., 1998) were mated for embryo collections. Late gastrulae were dissected (E6.75-7.0) for further analysis. All mouse studies were performed under a UK Home Office project license and complied fully with the UK Animals (Scientific Procedures) Act 1986 as implemented by the University of Cambridge and the Medical Research Council. Embryos were fixed for 20-30 min at room temperature in 4% paraformaldehyde (PFA) supplemented with 0.1% Tween-20 (Sigma) and 0.01% Triton-X100 (Sigma). Immunostaining was performed following standard procedures as described in the methods in the supplementary material. Fluorescent images were captured using a Zeiss LSM 710 microscope.

## Supplementary Material

Supplementary Material
