# Supplementary material for: Brachyury and SMAD signalling collaboratively orchestrate distinct mesoderm and endoderm gene regulatory networks in differentiating human embryonic stem cells
Source: Development. 2015 Jun 15;142(12):2121–35. doi: 10.1242/dev.117838 (PMC4483767; doi:10.1242/dev.117838)
Supplement: Supplementary Material [file supp_142_12_2121__index.html]

Brachyury and SMAD signalling collaboratively orchestrate distinct mesoderm and endoderm gene regulatory networks in differentiating human embryonic stem cells — Supplementary Material 

# Brachyury and SMAD signalling collaboratively orchestrate distinct mesoderm and endoderm gene regulatory networks in differentiating human embryonic stem cells

## DEV117838 Supplementary Material

- Supplementary Material
