# Supplementary material for: Brachyury and SMAD signalling collaboratively orchestrate distinct mesoderm and endoderm gene regulatory networks in differentiating human embryonic stem cells
Source: Development. 2015 Jun 15;142(12):2121–35. doi: 10.1242/dev.117838 (PMC4483767; doi:10.1242/dev.117838)
Supplement: Supplementary Material [file supp_dev.117838_DEV117838supp.pdf]

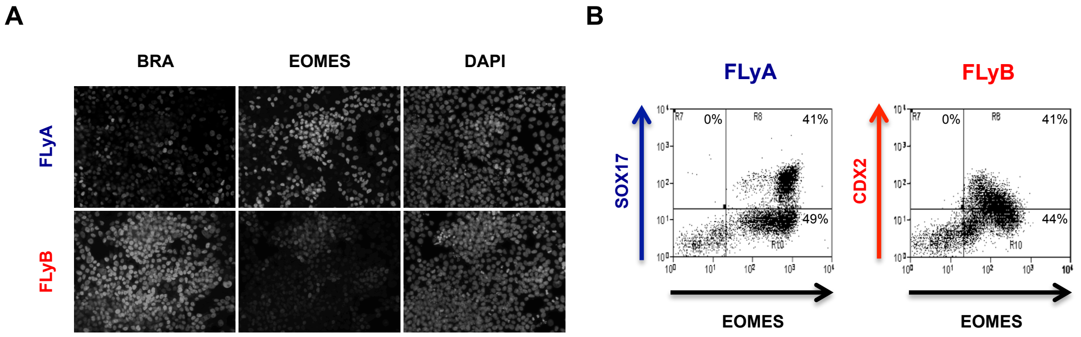

Fig. S1. An *in vitro* differentiation system to study the role of BRACHYURY in human gastrulation. (A) Fluorescent microscopy analysis of hESCs differentiated in FLyA or FLyB media for 36 hours. Cells were immunostained for BRA and EOMES. Nuclei were stained with DAPI. (B) Flow cytometry analysis of hESCs differentiated in FLyA or FLyB media for 36 hours. FLyA-treated cells were co-immunostained for SOX17 and EOMES (left). FLyB-treated cells were co-immunostained for CDX2 and EOMES (right).

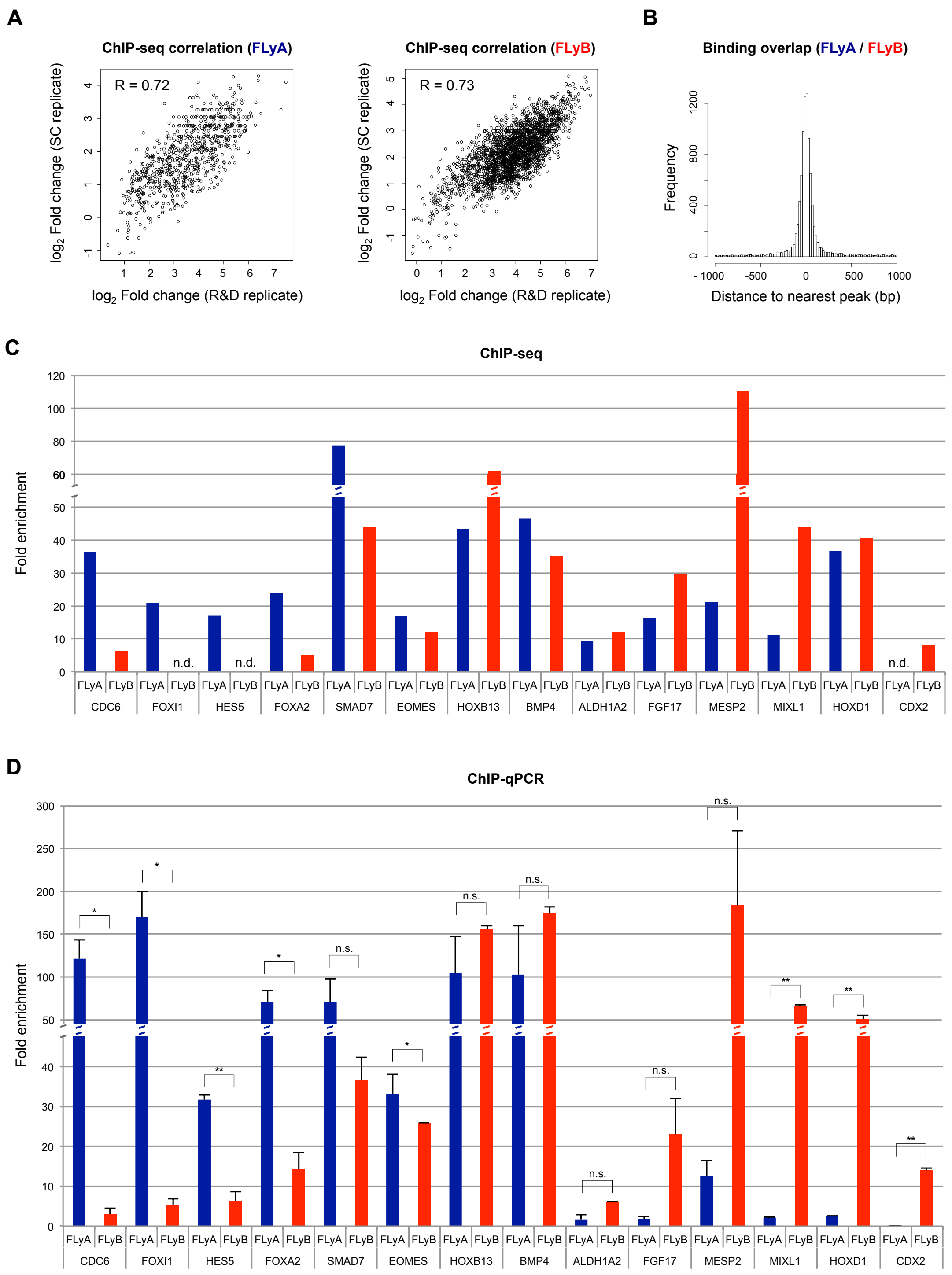

Fig. S2. BRACHYURY exhibits distinct genomic binding profiles in FLyA- or in FLyB-differentiated hESCs. (A) Dot plots showing ChIP-seq fold enrichment values (ChIP over input) of BRA peaks detected in both ChIP-seq replicates, obtained from either FLyA-treated hESCs (left) or FLyB-treated hESCs (right). SC – Santa Cruz antibody, R&D – R&D Systems antibody, R – correlation coefficient. (B) Histogram showing the distance between BRA-binding peaks in FLyA- and FLyB-treated hESCs. (C) and (D) Graphs showing fold enrichment values (ChIP over input) for BRA binding in FLyA (blue) or FLyB (red) conditions as assessed by ChIP-seq (upper graph) or ChIP-qPCR (lower graph), as indicated. BRA target genes are shown

at the bottom of the graphs. Error bars correspond to standard deviation (n = 3) (Student's two-tailed t-test: * $p < 0.05$; ** $p < 0.01$; n.s. – not significant). ChIP-qPCR values were normalized to a control IgG. n.d. – no data.

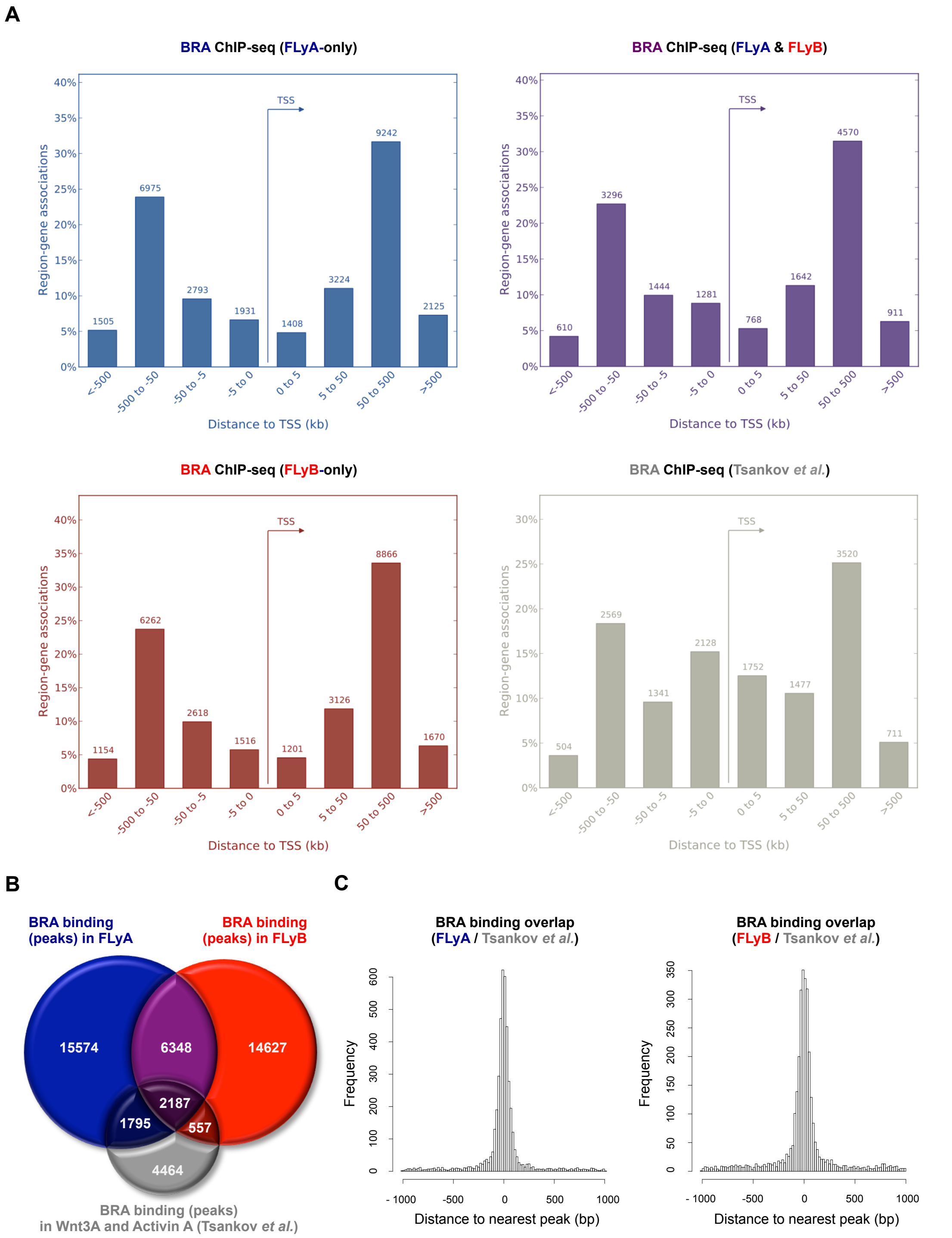

Fig. S3. Analysis of BRA genomic binding in hESCs differentiated using different conditions. (A) Spatial distribution of BRA binding relative to the transcription start sites (TSS) of its putative target genes in FLyA-only peaks (top left), in FLyA and FLyB common peaks (top right), in FLyB-only peaks (bottom left) and in Wnt3A/Activin peaks (Tsankov et al., 2015) (bottom right); graphs were generated using GREAT (McLean et al., 2010) with default settings. (B) Venn diagram showing the detectable overlap between BRA binding (ChIP-seq peaks) in FLyA-treated hESCs (blue), FLyB-treated hESCs (red), and Wnt3A/Activin-treated hESCs (Tsankov et al., 2015) (grey). (C) Histograms showing the distance between BRA-

binding peaks in Wnt3A/Activin-treated hESCs (Tsankov et al., 2015) and FLyA-treated hESCs (left) or FLyB-treated hESCs (right).

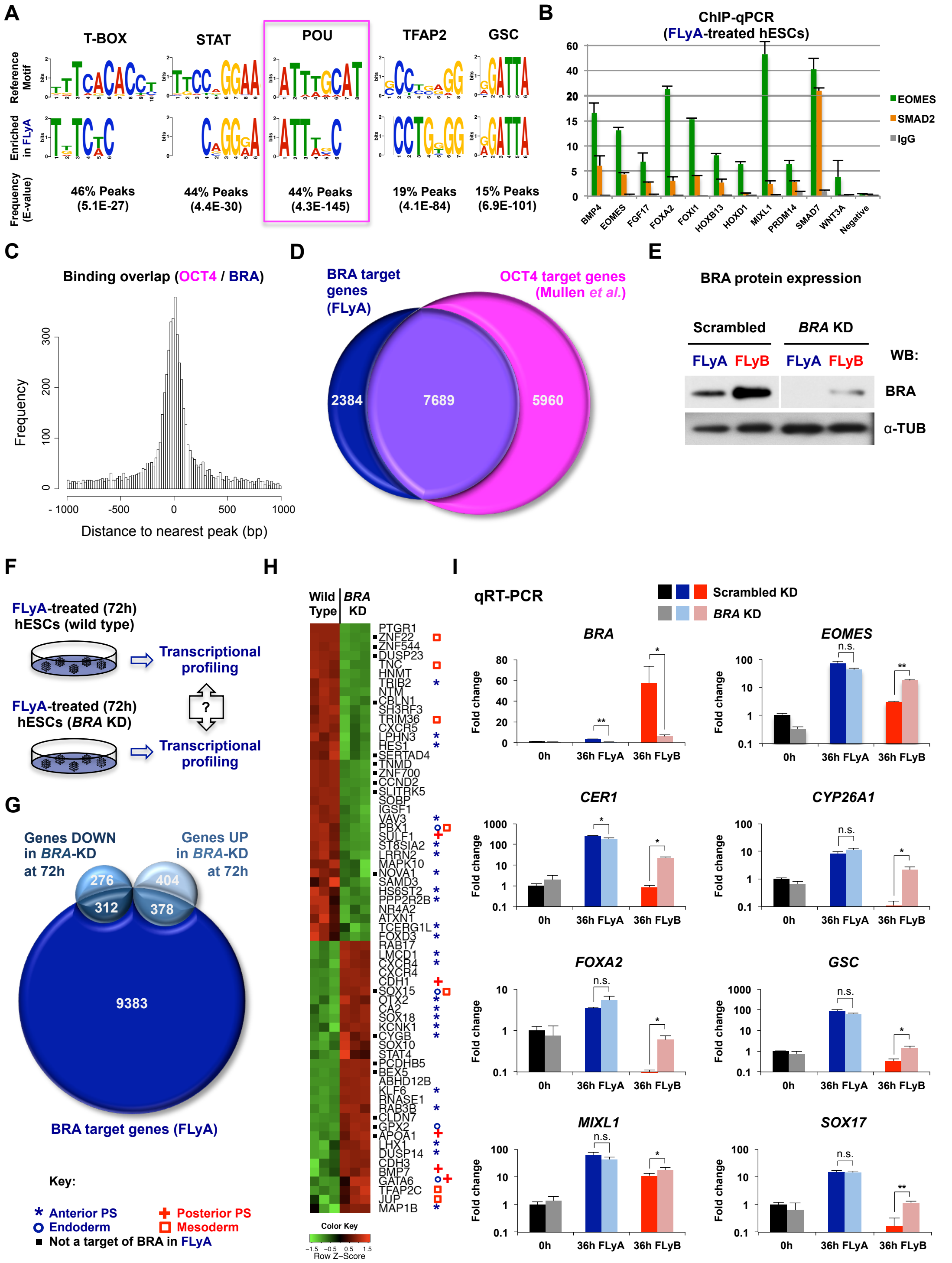

Fig. S4. BRACHYURY in the context of Activin A signalling. (A) Comparison of DNA recognition sites of five protein families (row above) and DNA motifs enriched in BRA FLyA ChIP-seq peaks (below). (B) Graph with fold enrichment values (ChIP over input) for EOMES binding (green), SMAD2 binding (orange) and control IgG binding (grey) to BRA target regions in FLyA-treated hESCs (36 hours). Error bars correspond to standard deviation (n = 3). ChIP-qPCR values were normalized to the highest control IgG value (*SMAD7*). (C) Histogram showing the distance between BRA-binding peaks in FLyA-treated hESCs and OCT4 binding in pluripotent hESCs (Mullen et al., 2011). (D) Venn diagram showing the overlap between BRA putative

target genes (FLyA, blue) and OCT4 binding in pluripotent hESCs (Mullen et al., 2011; magenta). (E) Western blot analysis of protein extracts from FLyA- or FLyB-treated (36 hours) hESCs transfected with a mock shRNA (Scrambled) or *BRA* shRNA (*BRA* KD). Membranes were blotted for BRA and α-TUBULIN (loading control). 10 µg of total protein were loaded in each lane. (F) hESCs (wild type or *BRA* knockdown) were differentiated for 72 hours in FLyA medium and transcriptionally compared. (G) Venn diagram showing the overlap between BRA putative target genes (FLyA, dark blue) and genes that were either up- or down-regulated at 72 hours (FDR < 0.05) in *BRA* knockdown hESCs when compared to wild type hESCs. (H) Microarray gene expression heat map of wild type versus *BRA* knockdown (KD) hESCs grown in FLyA for 72 hours. Selected genes were those that showed the greatest misregulation (up- or down-regulated) in *BRA* knockdown cells. A few additional genes were included for their biological relevance. Green indicates down-regulation and red indicates up-regulation. Symbols after gene names indicate expression pattern *in vivo* (Mouse Genome Informatics; Alev et al., 2010): **+** / □ - posterior primitive streak / mesoderm; ***** / **o** - anterior primitive streak / endoderm. (I) Quantitative RT-PCR of BRA target genes expressed in endoderm or anterior primitive streak. hESCs transfected with either a scrambled shRNA vector (dark colours) or a shRNA against *BRA* (*BRA* KD; light colours) were differentiated as indicated. “0h” are samples from pluripotent cells. Bars indicate standard deviation ($n = 3$) (Student’s two-tailed t-test: * $p < 0.05$; ** $p < 0.01$; n.s. - not significant).

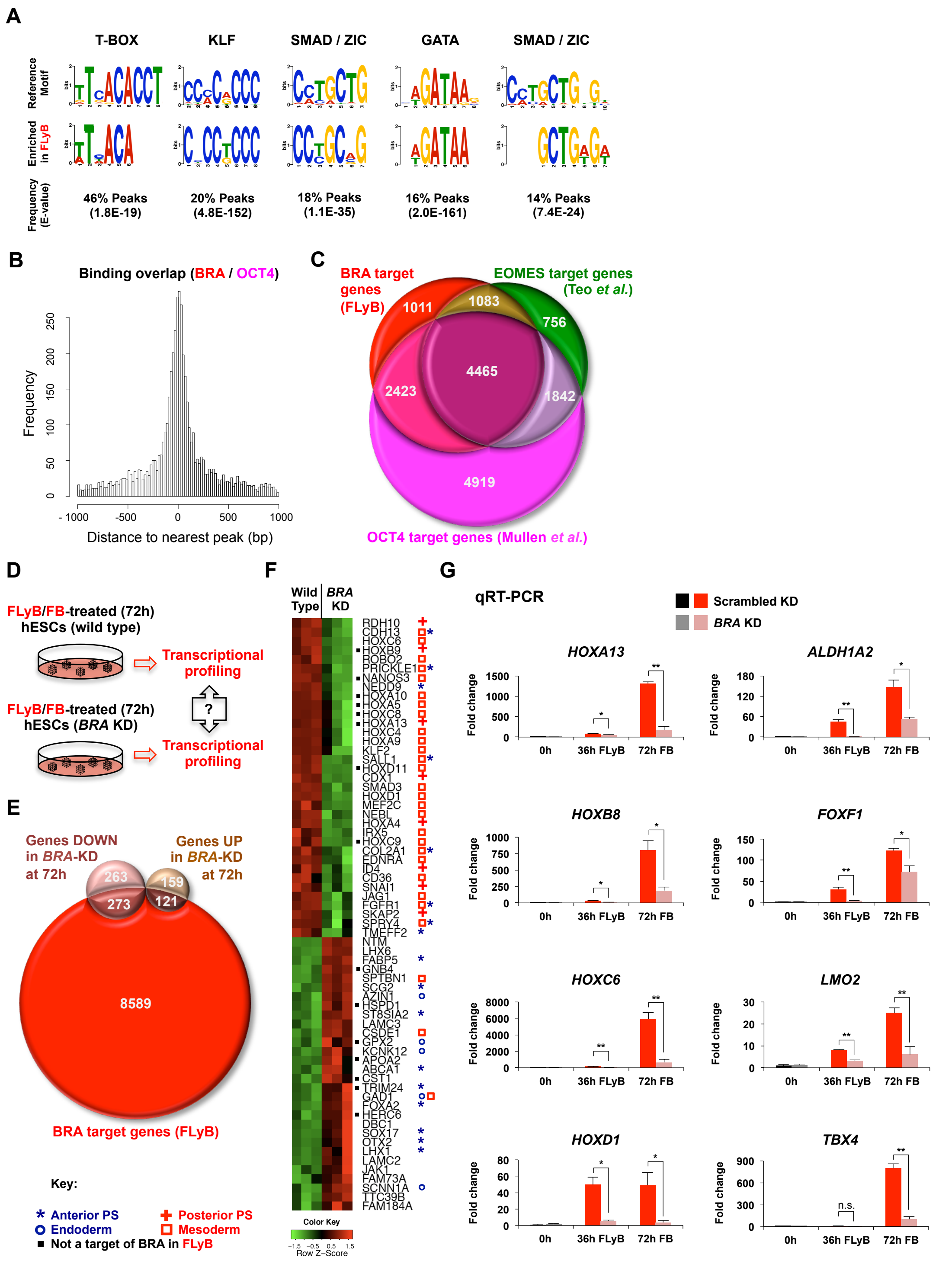

Fig. S5. BRACHYURY in the context of Bmp4 signalling. (A) Comparison of DNA recognition sites of five protein families (row above) and DNA motifs enriched in BRA FLyA ChIP-seq peaks (below). (B) Histogram showing the distance between BRA-binding peaks in FLyB-treated hESCs and OCT4 binding in pluripotent hESCs (Mullen et al., 2011). (C) Venn diagram showing the overlap between BRA putative target genes (FLyB, red), EOMES (Teo et al., 2011; green) and OCT4 binding in pluripotent hESCs (Mullen et al., 2011; magenta). (D) hESCs (wild type or *BRA* knockdown) were differentiated for 36 hours in FLyB medium followed by another 36 hours in FB medium (FLyB/FB; Bernardo et al., 2011) and transcriptionally

compared. (E) Venn diagram showing the overlap between BRA putative target genes (FLyB, red) and genes that were either up- or down-regulated (FDR < 0.05) in *BRA* knockdown hESCs (*BRA* KD) when compared to wild type hESCs, both differentiated in FLyB/FB media for 72 hours. (F) Microarray gene expression heat map of wild type versus *BRA* knockdown (*BRA* KD) hESCs grown in FLyB/FB media for 72 hours. Selected genes were those that showed the greatest misregulation (up- or down-regulated) in *BRA* knockdown cells. A few additional genes were included for their biological relevance. Green indicates down-regulation and red indicates up-regulation. Symbols after gene names indicate expression pattern *in vivo* (Mouse Genome Informatics; Alev et al., 2010): **+** / □ - posterior primitive streak / mesoderm; ***** / **o** - anterior primitive streak / endoderm. (G) Quantitative RT-PCR of BRA target genes expressed in mesoderm or posterior primitive streak. hESCs transfected with either a scrambled vector (dark colours) or a shRNA against *BRA* (*BRA* KD; light colours) were differentiated as indicated. “0h” are samples from pluripotent cells. Bars indicate standard deviation (n = 3) (Student’s two-tailed t-test: * $p < 0.05$; ** $p < 0.01$; n.s. - not significant).

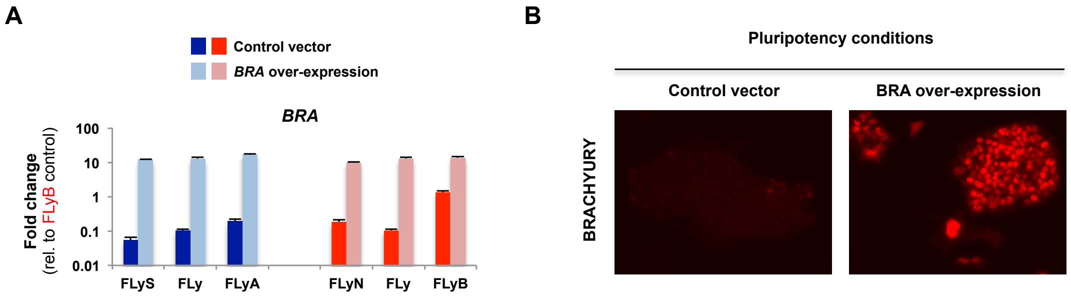

Fig. S6. BRACHYURY over-expression in hESCs. (A) Quantitative RT-PCR of *BRA* transcripts. hESCs transfected with either an empty/control vector (dark colours) or a *BRA* over-expression vector (light colours) were differentiated as indicated in Figure 6A and Figure 6B. Bars indicate standard deviation (n = 3). (B) Fluorescent microscopy analysis of H9 hESCs subclones transfected with either an empty/control vector or a *BRA* over-expression vector and grown in pluripotency conditions and treated with 1 μM 4-hydroxytamoxifen for 2h. Samples were immunostained for BRACHYURY. Abbreviations: Fgf2 (F), Ly294002 (Ly), Bmp4 (B), Activin A (A), Noggin (N), and SB431542 (S).

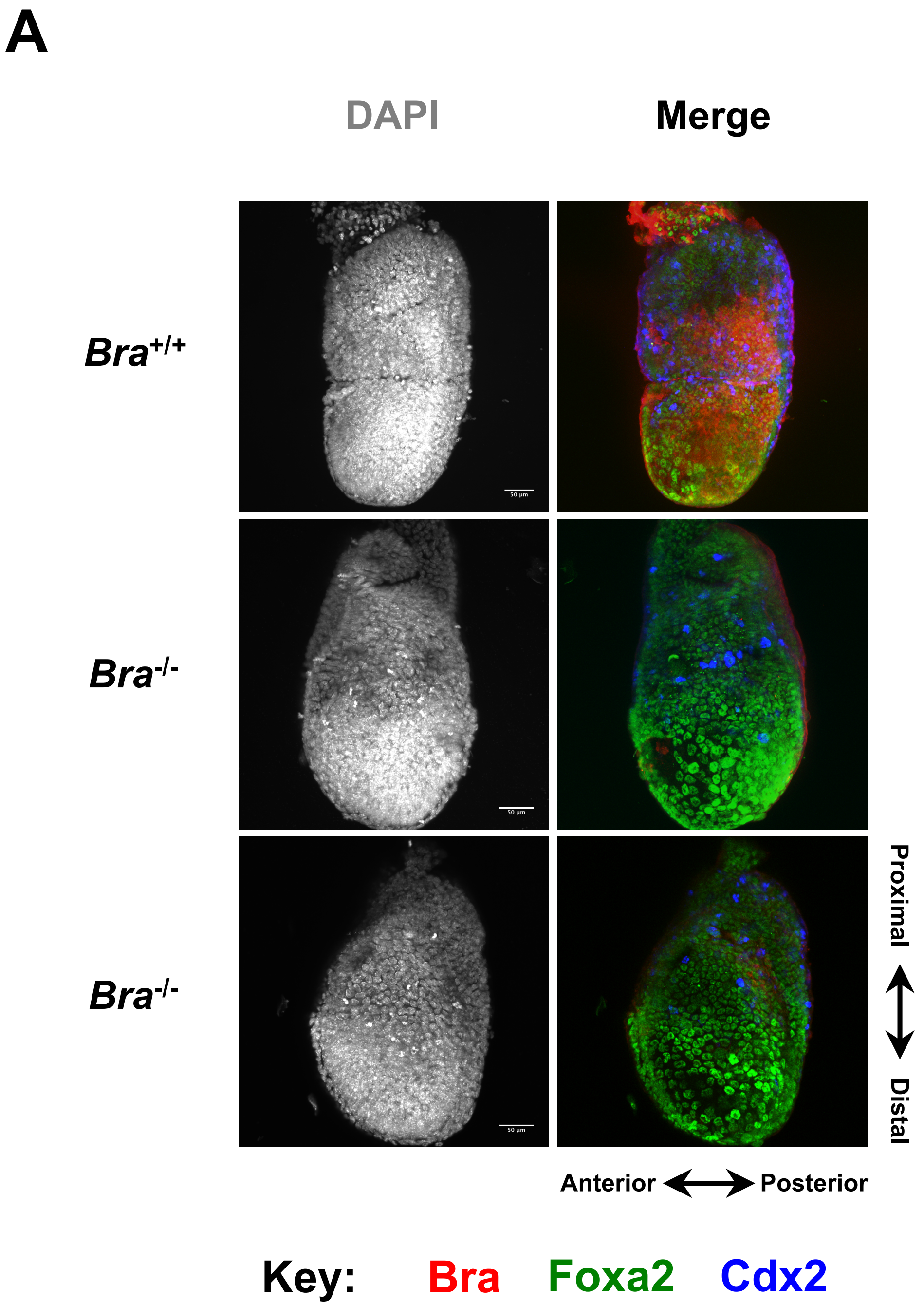

Fig. S7. Gene expression analysis of mouse gastrulae (wild type and *Bra* mutants) (A) Confocal microscopy analysis (maximal projection) of mouse gastrulae (E7.0) immunostained for Brachyury (red), Foxa2 (green) and Cdx2 (blue) (right column, merged pictures). Nuclei were stained with DAPI (left column). Upper row shows a wild type embryo (*Bra* $^{+/+}$). Bottom rows show *Brachyury* mutant embryos (*Bra* $^{-/-}$).

SUPPLEMENTARY METHODS

Human ESC Culture in Chemically Defined Conditions

Cells were differentiated as described in Bernardo et al., 2011.
H9 hESCs were cultured in a chemically-defined medium (CDM) supplemented with Fgf2 (20 ng/ml for differentiation and 12 ng/ml in pluripotency conditions) (recombinant zebrafish protein, made in the laboratory of Dr. Marko Hyvönen, University of Cambridge), 10 µM LY294002 (Sigma), 200 ng/ml Noggin (R&D Systems), 10 µM SB431542 (Tocris), 10 ng/ml (FLyB) or 50 ng/ml (FB) Bmp4 (R&D Systems) and either 10 ng/ml (pluripotency) or 100 ng/ml (FLyA) Activin A (made in the laboratory of Dr. Marko Hyvönen, University of Cambridge). Posterior (lateral plate) mesoderm differentiation was induced by treating cells with FLyB for 36 hours followed by Fgf2 + Bmp4 (50 ng/ml) for another 36 hours. All experiments were repeated at least twice on different cell passages to ensure results were reproducible.

Human ESC knockdown and overexpression lines

Stable H9 hESC sub-clones of *BRA* knockdown or *BRA* over-expression were established using a pLKO.1-puro shRNA vector (Sigma) or a pTP6 BRA-ER^{T2} vector, respectively. Briefly, the pTP6 BRA-ER^{T2} vector was generated by using the pTP6 PDX1-VP16-ER^{T2} vector (Bernardo et al., 2009) and replacing the *PDX1-VP16* sequence with the *BRA* coding sequence (*BRA* ORF cDNA, Origene, RC218530).
A Scrambled pLKO.1-puro shRNA vector (Sigma) was used as a control for the knockdown experiments whereas an "empty" pTP6 ER^{T2} vector was used as a control for the over-expression experiments.
All vectors were introduced into H9 cells using Lipofectamine 2000 (Invitrogen) transfection following the manufacturer's instructions.
After transfecting the vectors, colonies were allowed to grow for 24-48 hours before the addition of 1 µg/ml puromycin to the cultures. After approximately one week of antibiotic treatment, surviving colonies (sub-clones) were manually picked and expanded for further analyses. In the over-expression experiments, 1 µM 4-hydroxytamoxifen (Sigma) was added to the cultures in the beginning of the differentiation protocol and maintained for 36 hours. Puromycin (Sigma) was added to the media in all experiments involving vector-transfected cells.
Stable clones were screened by qRT-PCR and immunostaining (BRA expression) and compared to control transfected lines.

Flow cytometry

Cells were briefly washed with PBS and incubated for 5 minutes in Cell Dissociation Buffer (Gibco, 13150-016) at room temperature. After an additional wash in PBS, the suspended cells were fixed using the Cytofix/Cytoperm kit (BD Biosciences, 555028) following the manufacturer's instructions.
After fixation, the antibodies directly conjugated to a fluorophore were added first: anti-BRACHYURY coupled to Phycoerythrin (R&D Systems, IC2085P) and anti-SOX17 coupled to Allophycocyanin (R&D Systems, IC1924A). The antibodies that were not directly conjugated to a fluorophore [mouse anti-CDX2 (Biogenex, MU392A-UC) and rabbit anti-EOMES (abcam, ab23345)] were added subsequently at a 1/200 dilution. This was followed by the addition of donkey secondary antibodies conjugated to Alexa Fluor 488 (donkey anti-mouse, or donkey anti-rabbit at a 1/400 dilution) (Invitrogen). Where required, an intermediate blocking step was performed.

Each antibody incubation was done at 4°C for 30 minutes in blocking buffer (made in Cytoperm buffer) and was followed by 3 washes in Cytoperm buffer. Lastly, cells were re-suspended in PBS supplemented with 1% BSA.
The appropriate isotype controls, primary antibody omission controls and cells-only controls, as well as single positive samples for each channel were included as controls.

Immunofluorescence
Cells were fixed for 15 minutes at room temperature in 4% paraformaldehyde (PFA) (Sigma) and then washed three times with PBS. Cells were blocked and permeabilised with PBS containing 10% donkey serum (Serotec) and 0.1% Triton-X (Sigma) for 30 minutes at room temperature.
Subsequently, cells were incubated overnight at 4°C with primary antibodies diluted 1/100 in PBS with 1% donkey serum [goat anti-BRACHYURY (R&D Systems, AF2085) and rabbit anti-EOMES (abcam, ab23345)].
Cells were then washed three times with PBS and incubated with the appropriate secondary antibody (Alexa Fluor, Invitrogen) diluted 1/300 in PBS with 1% donkey for 1-2 hours at room temperature. The cells were washed three times with PBS. Nuclear stain was provided by adding DAPI in the first wash at a 1/10000 dilution.

Endogenous Protein Co-Immunoprecipitation
All buffers contained 20 mM Hepes, 0.2 mM PMSF, 0.5 mM EDTA, Complete protease inhibitor mix (Roche), 0.2 mM DTT and varying concentrations of Glycerol (G), KCl (K) or Sucrose (S), where K20 stands for 20 mM KCl, N50 for 50 mM NaCl, S250 for 250 mM Sucrose, etc.
Cells were harvested in Cell Dissociation Buffer (Gibco) and lysed in ice-cold K20G5 using a Dounce homogenizer with 20 strokes. Nuclei were washed in isotonic K20S250, re-suspended in two volumes of K10G20 and extracted by addition of 400mM NaCl, douncing and subsequent rotation for 1h at 4°C. Chromatin and debris were separated from the nuclear extract (supernatant) by ultracentrifugation at 12000g and then dialyzed against K100N50 to establish near physiological salt conditions. Co-immunoprecipitation was carried out with antibodies indicated in the figure legends [R&D systems: SMAD1 (AF2039), SMAD2/3 (AF3797), control IgG (AB-108-C)] followed by ProteinG beads (Roche) and final washes in K100G10+0.02%Tween20. Samples were subsequently eluted in LDS sample buffer (Invitrogen) and run on a SDS polyacrylamide gel in preparation for analysis by immunoblotting. Volumes loaded: 5%-10% (Input), 50% (Immunoprecipitate).

Western blotting
Protein samples were loaded onto 4-12% sodium dodecyl sulphate-polyacrylamide gels (SDS-PAGE). Electrophoresis was carried out with an XCell SureLock Minicell system (Invitrogen) according to the manufacturer's instructions, using NuPAGE MOPS running buffer (Invitrogen). Samples were then transferred to PVDF membranes (Millipore). Immunodetection was performed according to manufacturer's instructions (Millipore) using the following primary antibodies (1/1000-1/5000 dilution): BRACHYURY (R&D Systems, AF2085), EOMESODERMIN (abcam, ab23345), SMAD1 (R&D Systems, AF2039), SMAD2/3 (R&D Systems, AF3797), Phospho-SMAD1/5/9 (Cell Signalling, 13820), Phospho-SMAD2/3 (Cell Signalling 8828), β-ACTIN (Sigma, A3853), and α-TUBULIN (abcam, ab7291) in 5% non-fat dry milk dissolved in TBS-T (150 mM sodium

chloride, 10 mM Tris pH8.0, and 0.1% Tween 20 dissolved in water). Detection was carried out with secondary ExactaCruz reagents (Santa Cruz) for Co-IP samples or secondary HRP-conjugated antibodies at 1/10000 dilution (SIGMA) for normal Western blots. Dura-West substrate (Thermo Scientific) was used to develop the immunoblots prior to X-ray film exposure, following the manufacturer's instructions.

Total RNA extraction

Cells were washed briefly with PBS before adding cell lysis buffer (RLT buffer, QIAGEN). Total RNA was then extracted using the RNeasy Mini kit (QIAGEN) following the manufacturer's instructions. During RNA purification, spin columns were incubated with RNase-free DNase (QIAGEN, 79254) to remove residual DNA. After washing, the purified RNA was eluted in 30 μl of DEPC-treated water (Ambion). Samples were assessed for RNA concentration and quality using a NanoDrop spectrometer. Purified RNA was stored at -80ºC for further analyses.

Sample preparation for Illumina BeadArrays

Total RNA was extracted from cultured cells using the RNeasy Mini Kit (QIAGEN) as described above. RNA was then amplified, biotin labelled, and hybridized to Human WG-6 expression BeadArrays (Illumina) using a BeadScanner/BeadStation (Illumina) according to the manufacturer's standard protocols (Illumina) at the Microarray Resources Centre, Department of Pathology, University of Cambridge. Three biological replicates were hybridized for each experimental condition.

qRT-PCR

Quantitative reverse transcription polymerase chain reaction (qRT-PCR) mixtures were prepared using Fast SYBR Green Master Mix (Applied Biosystems, 4385614), according to the manufacturer's instructions. PCR reactions were performed in a 7500 Fast Real-Time PCR System (Applied Biosystems) following the manufacturer's instructions.

Three μl of diluted cDNA (1/200) from each sample were analysed in duplicate wells for specific gene expression. Expression values were then normalized by the expression of the "house-keeping" gene *Porphobilinogen Deaminase* (*PBGD*) in each sample, which was analysed in the same PCR plate. Relative gene expression was assessed using the formula $2^{-\Delta Ct}$, where the Ct value corresponds to the cycle (within the exponential phase of the PCR reaction) at which the fluorescence crosses the optimal threshold established by Applied Biosystems software before DNA concentration begins to plateau. The ΔCt is the difference between the Ct of the gene of interest and the Ct of *PBGD*.

Error bars on all qPCR graphs represent standard deviation from three independent biological replicates. Student's t-tests (two-tailed assuming non-equal variance) were performed. Primer sequences can be found in Table S6.

Chromatin immunoprecipitation (ChIP)

ChIP was performed as previously described (Brown et al., 2011) with some modifications.

Human ESCs (between 2 x10^7 and 5 x 10^7) were grown in 10 cm plates and differentiated in FLyA or FLyB conditions as previously described. Before crosslinking, the growth medium was aspirated and the cells were rapidly washed with PBS. Crosslinking was then carried out in two steps: 1) treating the cells with PBS supplemented with 10 mM Dimethyl 3,3′-dithiopropionimidate dihydrochloride

(DTBP) (Sigma, D2388) and 2.5 mM 3,3′-Dithiodipropionic acid di(N-hydroxysuccinimide ester) (DSP) (Sigma, D3669) at room temperature for 15 minutes; 2) adding 1% formaldehyde (Sigma) and incubating it at room temperature for another 15 minutes. The crosslinkers were then neutralized by adding 0.125 M Glycine and incubating at room temperature for 5 minutes.

Cells were then rinsed with cold PBS and harvested in cold PBS supplemented with protease inhibitors (Roche, 11873580001), 50 μg/ml PMSF (Sigma, 93482) and 1 μg/ml Leupeptin (Roche, 11017128001) using a cell scraper. The cells were resuspended in 2 ml of Cell Lysis Buffer (10 mM Tris pH 8.0, 10 mM NaCl, 0.2% NP-40) and incubated for 10 minutes on ice. After centrifugation (1800 rpm at 4ºC for 5 minutes), the nuclei were resuspended in 1.25 ml of Nuclear Lysis Buffer (50 mM Tris pH 8.1, 10 mM EDTA, 1% SDS) and incubated for 10 minutes on ice.

The nuclear lysate was then diluted by adding 0.75 ml of IP Buffer (20 mM Tris-HCl pH8.0, 2 mM EDTA, 150 mM NaCl, 0.01% SDS, 1% Triton x-100) before sonication.

The chromatin was sonicated using a Misonix 4000 to obtain DNA fragments between 200bp and 400bp in size, and the samples were subsequently diluted to a 5.5 ml final volume with IP Buffer. An "Input" sample (300 μl) was taken before immunoprecipitation for later processing.

The samples were then incubated overnight at 4°C with 10 µg of antibody (specific or non-specific). Antibodies used: BRACHYURY (R&D Systems, AF2085), BRACHYURY (Santa Cruz, sc-20109), EOMESODERMIN (abcam, ab23345), SMAD2 (Cell Signaling, 5339S), SMAD1 (Cell Signaling, 9743S), control IgGs (Santa Cruz).

One hundred μl magnetic Protein G Dynabeads (Invitrogen) were used per ChIP. The beads were previously washed with PBS, blocked overnight with 0.5% BSA in PBS and then added to the samples. After 4 hours of rotation at 4°C, the bead-chromatin complexes were retrieved using a magnetic rack (Invitrogen).

The beads were then washed twice with Wash Buffer 1 (20 mM Tris pH8.0, 2 mM EDTA, 50 mM NaCl, 0.1% SDS, 1% Triton X-100), once with Wash Buffer 2 (10 mM Tris pH8.0, 1 mM EDTA, 0.25 M LiCl, 1% NP-40, 1% Deoxycholic acid, sodium salt), and twice with TE Buffer (10 mM Tris pH8.0, 1 mM EDTA). The chromatin was then eluted twice (15 minutes each) in 150 μl of Elution Buffer (100 mM $NaHCO_3$, 1% SDS) using a thermomixer (Eppendorf) at 65°C (1000 rpm).

To reverse the crosslinking and remove RNAs, the samples (ChIP and Input) were treated with 100 mM DTT (Fluka, 43819) for 30 minutes at 37°C, followed by treatment with 1 µl RNase A (1 mg/ml) (Invitrogen) and 18 µl 5 M NaCl at 65°C for 8-12 hours. To remove all the proteins and recover the DNA, samples were treated with 3 µl Proteinase K (20 mg/ml) (Roche) for 2 hours at 45°C, followed by phenol/chloroform DNA extraction and ethanol precipitation using GlycoBlue (Ambion). Finally, the DNA was resuspended in 50 µl (ChIP samples) or 100 µl (Input samples) of nuclease-free water (Ambion) for further analyses.

Preparation of Illumina ChIP-seq libraries

Preparation of ChIP-seq libraries was carried out using the ChIP-seq DNA sample kit (Illumina, IP-102-1001) according to manufacturer's instructions.

Minor modifications included: 1) using 5 ng of DNA as starting material [DNA concentration was measured using a Qubit fluorometer (Invitrogen) according to the manufacturer's instructions], 2) diluting the Illumina adaptor mix 40x before use, as recommended by Schmidt et al. (Schmidt et al., 2009), 3) using the DNA Clean &

Concentrator-5 kit (Zima Research) throughout the protocol, 4) performing the final size selection of DNA fragments (200bp-250bp) using the E-gel SizeSelect (2% agarose) system (Invitrogen).

Sequencing of Illumina ChIP-seq libraries

Processing of the samples was carried out at the MRC National Institute for Medical Research in London. The quality assessment of the libraries was carried out using the Agilent 2100 Bioanalyser to verify 1) the absence of primer dimers and 2) that the selected DNA fragments had a single-peak distribution with the desired size (around 200 bp). The libraries were finally sequenced in a Genome Analyzer (Illumina), according to manufacturer's instructions.

ChIP-qPCR

One μl of eluted DNA from each ChIP sample was analysed in duplicate and normalized to the Input. Target enrichment was assessed using the formula $(1+\text{Primer Efficiency})^{-\Delta Ct}$, as previously described (Aparicio et al., 2004; Mukhopadhyay et al., 2008). The ΔCt is the difference between the Ct of the ChIP sample and the Ct of the Input sample. Primer sequences can be found in Table S6.

Illumina BeadArray analysis – Data processing

Data provided in raw bead-level format was imported into the R statistical programming environment (http://www.r-project.org/). The background was corrected with the RMA algorithm (Irizarry et al., 2003) and summarized via the BeadArray package of the Bioconductor (http://www.bioconductor.org) suite of open-source bioinformatics software. Once imported and processed as described, profiles were $\log_2$ transformed and quantile normalised using the Bioconductor:limma package.

Illumina BeadArray analysis – Differential expression

For each microarray probe-set, significant difference in expression between groups of sample profiles, including those from Bernardo et al. (2011) (FLyA- or FLyB-treated hESCs, 36 hours), was assessed using the moderated t-statistic of (Wettenhall and Smyth, 2004), as implemented in Bioconductor:limma. To correct for multiple hypothesis testing on such a scale, significance p-values obtained for all probe-sets were corrected using the false discovery rate (FDR) method of Storey and Tibshirani (2003) and differential expression deemed significant at a false discovery rate (FDR) of 5% ($q < 0.05$). Shared EntrezGene IDs were used to quantify intersection between differentially regulated genes and BRACHYURY-bound regions (see ChIP-seq methods) and to create corresponding Venn diagrams.

Illumina BeadArray analysis – Data visualization

Heatmaps of comparative gene expression were created via application of the heatmap.2 method from Bioconductor:gplots to gene expression profiles as described above. For each probe-set, $\log_2$ expression intensities were scaled to have zero mean and unit standard deviation across all samples present. In the case wherein a gene was represented by more than one microarray probe-set, a single probe-set was selected for display according to highest mean expression across all samples regardless of sample group (highest average signal).

Illumina BeadArray analysis – Gene ontology analysis

For all differentiation conditions (FLyA at 36 hours, FLyA at 72 hours, FLyB at 36 hours, FLyB-FB at 72 hours), lists of BRA target genes that were either up- or down-regulated (FDR < 0.05; Fold change > 2.0) in *BRA* knockdown cells as compared to wild type cells were uploaded to UCSC Genome Browser (https://genome.ucsc.edu) and used to create BED files using the Table Browser function. The resulting BED files containing "whole gene" coordinates were then uploaded onto GREAT (http://great.stanford.edu) and analysed using a "single nearest gene" approach (McLean et al., 2010). The threshold of statistical significance was $p < 1 \times 10^{-5}$.

Mouse RNA-seq analysis

Mouse Bra RNA-seq raw data (Lolas et al., 2014) were not re-analysed; FPKM (Fragments Per Kilobase of exon per Million fragments mapped) values reported in Lolas et al. were used for further analyses. Only differentially expressed (fold change > 2.0) genes were used for further analysis. Mouse/human gene homology analysis was performed using http://www.mousemine.org/.

ChIP-seq analysis – Data processing

The sequence tags resulting from Illumina sequencing were aligned to the human genome (hg19) with the Bowtie short-read aligner (Langmead et al, 2009). Settings allowed for a maximum of two mismatches (default setting) in an aligned sequence and only a unique alignment was retained for each tag**.** Remaining tags for both ChIP and Input samples were input to the MACS1.4 peak finder (Zhang et al, 2008) with the following settings (tsize = 35; mfold = 10,16; bw = 100; gsize = 2700000000). Different thresholds for peak detection were used for different ChIP-seq datasets: for BRA (R&D systems antibody), EOMES (Teo et al., 2011), SMAD2/3 (Brown et al., 2011), and OCT4 (Mullen et al., 2011), p-value was 1e-12; for BRA (Santa Cruz antibody), p-value was 1e-9; for BRA (Tsankov et al., 2015), p-value was 1e-5. Mouse Bra ChIP-seq raw data (Lolas et al., 2014) were not re-analysed; peaks described in Lolas et al. were used for further analyses. All sequencing data have been uploaded to the Gene Expression Omnibus (GEO; http://www.ncbi.nlm.nih.gov/geo/) data repository.

ChIP-seq analysis – Bound region annotation

Bound regions were associated to UCSC Gene IDs. If a peak's coordinates overlap a known promoter region, the gene belonging to that promoter is assigned. If the peak is not in a promoter region but is intragenic, it is assigned the gene that it directly overlaps. Otherwise, if intergenic, the peak is assigned the nearest gene either side within 50 kb. Therefore intergenic peaks can be assigned up to two genes.

ChIP-seq analysis – Peak visualization

Each wig density profile was normalized to the number of unique reads/tags given the bowtie output using the in-house script "normalise.pl". Wig files were then converted to BigWig format using the wigToBigWig utility (http://genome.ucsc.edu/goldenPath/help/bigWig.html) for uploading to the UCSC genome browser.

ChIP-seq analysis – Histograms

Summit coordinates for each of the samples were taken from the MACS peaks output. The distance between the peak summits between each pair of samples was calculated

in base pairs using the closestBed (-d option) utility from the BEDtools suite (version 2.13.3). Peak distance was filtered to 1000 base pairs each side of the summit. The histogram for the distances between the two datasets was plotted with R (version 2.12.1).

ChIP-seq analysis – Fold-change plots

Fold change values of the called peaks were taken from the MACS output for each sample. The IntersectBed utility from BEDTools suite was used to calculate the overlap between shared peaks. Plot visualization was done with R.

ChIP-seq analysis – DNA motifs

DNA motif analyses were performed using all sequences bound by BRA in either FLyA or FLyB conditions, as determined by MACS (Zhang et al., 2008). The sequences, which were 200 bp-long, comprised the centre of BRA peaks flanked by the adjacent 100 bp-long regions on both 5' and 3' sides. Genomic sequences corresponding to each peak centre were obtained via the Bioconductor:BSgenome package. All analyses were carried out using tools from the MEME suite (http://meme.nbcr.net/meme/intro.html) (Bailey et al., 2009) using default parameters: MEME-ChIP was used for motif enrichment analysis, and TOMTOM for identification of enriched motifs.

Mouse Embryo Work

Embryos were stained within 2 days of being fixed and following permeabilization with 0.5% Triton and a blocking step performed with 2% donkey serum. All washes were done with 0.1% Triton-X100 (Sigma).

Primary antibody incubations were performed overnight at 4°C as follows: goat anti-Brachyury (1:50, R&D Systems), mouse anti-Cdx2 (Cdx2-88, 1:50, Abcam), rabbit anti-Foxa2 (1:50, Cell Signaling). Fluorescently labelled secondary antibodies (IgG, 1:400) were added for 1 h at room temperature as follows: Alexa 594 donkey anti-goat, Alexa 488 donkey anti-rabbit, Alexa 647 donkey anti-mouse (Invitrogen). Nuclear staining was performed with DAPI for at least 2h and embryos were mounted in slow-fade reagent (Life technologies).

Supplementary References

Aparicio, O., Geisberg, J.V. and Struhl, K. (2004). Chromatin immunoprecipitation for determining the association of proteins with specific genomic sequences in vivo. *Curr. Protoc. Cell Biol.* **Chapter 17**, Unit 17.7.

Bailey, T.L., Boden, M., Buske, F.A., Frith, M., Grant, C.E., Clementi, L., Ren, J., Li, W.W. and Noble, W.S. (2009). MEME SUITE: tools for motif discovery and searching. *Nucleic Acids Res.* **37**, W202-W208.

Bernardo, A.S., Cho, C.H., Mason, S., Docherty, H.M., Pedersen, R.A., Vallier, L. and Docherty, K. (2009). Biphasic induction of Pdx1 in mouse and human embryonic stem cells can mimic development of pancreatic beta-cells. *Stem Cells* **27**, 341-351.

Bernardo, A.S., Faial, T., Gardner, L., Niakan, K.K., Ortmann, D., Senner, C.E., Callery, E.M., Trotter, M.W., Hemberger, M., Smith, J.C., Bardwell, L., Moffett, A. and Pedersen, R.A. (2011). BRACHYURY and CDX2 mediate BMP-induced differentiation of human and mouse pluripotent stem cells into embryonic and extraembryonic lineages. *Cell Stem Cell* **9**, 144-155.

Brown, S., Teo, A., Pauklin, S., Hannan, N., Cho, C.H., Lim, B., Vardy, L., Dunn, N.R., Trotter, M., Pedersen, R. and Vallier, L. (2011). Activin/Nodal signalling controls divergent transcriptional networks in human embryonic stem cells and in endoderm progenitors. *Stem Cells* **29**, 1176-1185.

Irizarry, R.A., Hobbs, B., Collin, F., Beazer-Barclay, Y.D., Antonellis, K.J., Scherf, U. and Speed, T.P. (2003). Exploration, normalization, and summaries of high density oligonucleotide array probe level data. *Biostatistics* **4**, 249-264.

Langmead, B., Trapnell, C., Pop, M. and Salzberg, S.L. (2009). Ultrafast and memory-efficient alignment of short DNA sequences to the human genome. *Genome Biol.* **10**, R25.

Lolas, M., Valenzuela, P.D., Tjian, R. and Liu, Z. (2014). Charting Brachyury-mediated developmental pathways during early mouse embryogenesis. *Proc. Natl. Acad. Sci. USA* **111**, 4478-4483.

McLean, C.Y., Bristor, D., Hiller, M., Clarke, S.L., Schaar, B.T., Lowe, C.B., Wenger, A.M. and Bejerano, G. (2010). GREAT improves functional interpretation of cis-regulatory regions. *Nat. Biotechnol.* **28**, 495-501.

Mukhopadhyay, A., Deplancke, B., Walhout, A.J. and Tissenbaum, H.A. (2008). Chromatin immunoprecipitation (ChIP) coupled to detection by quantitative real-time PCR to study transcription factor binding to DNA in Caenorhabditis elegans. *Nat. Protoc.* **3**, 698-709.

Schmidt, D., Wilson, M.D., Spyrou, C., Brown, G.D., Hadfield, J. and Odom, D.T. (2009). ChIP-seq: using high-throughput sequencing to discover protein-DNA interactions. *Methods* **48**, 240-248.

Storey, J.D. and Tibshirani, R. (2003). Statistical significance for genome wide studies. *Proc. Natl. Acad. Sci. USA* **100**, 9440-9445.
Tsankov, A.M., Gu, H., Akopian, V., Ziller, M.J., Donaghey, J., Amit, I., Gnirke, A. and Meissner, A. (2015). Transcription factor binding dynamics during human ES cell differentiation. *Nature* **518**, 344-349.

Wettenhall, J.M. and Smyth, G.K. (2004). limmaGUI: a graphical user interface for linear modeling of microarray data. *Bioinformatics* **20**, 3705-3706.

Zhang, Y., Liu, T., Meyer, C.A., Eeckhoute, J., Johnson, D.S., Bernstein, B.E., Nusbaum, C., Myers, R.M., Brown, M., Li, W. and Liu, X.S. (2008). Model-based analysis of ChIP-seq (MACS). *Genome Biol.* **9**, R137.

Table S1: ChIP-seq data analysis of BRA-bound regions (peak location and gene annotation)

Click here to Download Table S1

Table S2: Gene ontology analysis of BRA-bound regions

Click here to Download Table S2

Table S3: Microarray gene expression profiling of BRA knockdown hESCs compared with wild-type hESCs

Click here to Download Table S3

Table S4: Gene ontology analysis of microarray data

Click here to Download Table S4

Table S5: Comparison between BRA target genes and RNA-seq data from mouse *Bra*-null embryos

Click here to Download Table S5

Table S6: Primers used for qRT-PCR and ChIP-qPCR

Click here to Download Table S6
